# A flexible high-throughput cultivation protocol to assess the response of individuals’ gut microbiota to diet-, drug-, and host-related factors

**DOI:** 10.1093/ismeco/ycae035

**Published:** 2024-03-12

**Authors:** Janina N Zünd, Serafina Plüss, Denisa Mujezinovic, Carmen Menzi, Philipp R von Bieberstein, Tomas de Wouters, Christophe Lacroix, Gabriel E Leventhal, Benoit Pugin

**Affiliations:** Laboratory of Food Biotechnology, Institute of Food, Nutrition and Health, Department of Health Sciences and Technology, ETH Zürich, 8092 Zürich, Switzerland; Laboratory of Food Biotechnology, Institute of Food, Nutrition and Health, Department of Health Sciences and Technology, ETH Zürich, 8092 Zürich, Switzerland; Laboratory of Food Biotechnology, Institute of Food, Nutrition and Health, Department of Health Sciences and Technology, ETH Zürich, 8092 Zürich, Switzerland; Laboratory of Food Biotechnology, Institute of Food, Nutrition and Health, Department of Health Sciences and Technology, ETH Zürich, 8092 Zürich, Switzerland; PharmaBiome AG, 8952 Schlieren, Switzerland; Laboratory of Food Biotechnology, Institute of Food, Nutrition and Health, Department of Health Sciences and Technology, ETH Zürich, 8092 Zürich, Switzerland; PharmaBiome AG, 8952 Schlieren, Switzerland; PharmaBiome AG, 8952 Schlieren, Switzerland; Laboratory of Food Biotechnology, Institute of Food, Nutrition and Health, Department of Health Sciences and Technology, ETH Zürich, 8092 Zürich, Switzerland; PharmaBiome AG, 8952 Schlieren, Switzerland; Laboratory of Food Biotechnology, Institute of Food, Nutrition and Health, Department of Health Sciences and Technology, ETH Zürich, 8092 Zürich, Switzerland

**Keywords:** ex vivo, in vitro, microbial ecology, xenobiotics, fibers, personalized microbiota response, anaerobic chamber, 16S rRNA gene sequencing, short-chain fatty acids, fecal microbiota

## Abstract

The anaerobic cultivation of fecal microbiota is a promising approach to investigating how gut microbial communities respond to specific intestinal conditions and perturbations. Here, we describe a flexible protocol using 96-deepwell plates to cultivate stool-derived gut microbiota. Our protocol aims to address gaps in high-throughput culturing in an anaerobic chamber. We characterized the influence of the gas phase on the medium chemistry and microbial physiology and introduced a modular medium preparation process to enable the testing of several conditions simultaneously. Furthermore, we identified a medium formulation that maximized the compositional similarity of *ex vivo* cultures and donor microbiota while limiting the bloom of *Enterobacteriaceae*. Lastly, we validated the protocol by demonstrating that cultivated fecal microbiota responded similarly to dietary fibers (resistant dextrin, soluble starch) and drugs (ciprofloxacin, 5-fluorouracil) as reported *in vivo.* This high-throughput cultivation protocol has the potential to facilitate culture-dependent studies, accelerate the discovery of gut microbiota-diet-drug-host interactions, and pave the way to personalized microbiota-centered interventions.

## Introduction

The human gut is inhabited by a diverse and dynamic assembly of microbes unique to each individual [1]. This microbial community contributes to key health functions such as nutrient metabolism, pathogen colonization resistance, gut barrier integrity maintenance, and host immune system education [2]. Despite advances in understanding the human gut microbiota’s diversity and functionality, the mechanisms governing ecological and physiological dynamics in response to specific intestinal conditions and perturbations remain poorly understood. Numerous studies have documented changes in the gut microbial community in response to dietary interventions [3–5], antibiotic/non-antibiotic medications [6–8], or alterations of the host physiology (e.g. intestinal pH, oxidative stress, osmotic pressure, antimicrobial peptides) [9–11]. However, high inter- and intra-individual variability of the gut microbiota, combined with the complex interactions between diet-, drug-, and host-related factors, has limited our capacity to generalize microbial responses to a broader population [12].

*Ex vivo* fecal cultivation holds promise for investigating the direct impact of relevant intestinal conditions on individuals’ gut microbiota in a controlled manner. Different setups have been employed for cultivating intestinal communities, ranging from simple batch incubation using anaerobic tube-based techniques [13–15] or multiwell plates [16–20] to a fully controlled, continuous multistage model [21–23]. Although continuous models more closely mimic *in vivo* intestinal conditions and enable more fundamental studies, they are labor-intensive, time-consuming, and limited by the number of donor microbiota and conditions that can be evaluated simultaneously. Further, many responses and activities are specific to donors [24–27], strains [28–31], and the physicochemical environment of the gut [9–11]. Cultivation with a higher throughput is thus crucial to advance our understanding of gut microbiota-diet-drug–host interactions and facilitate the development of personalized microbiota-centered interventions [4, 14]. Different high-throughput cultivation methods have been developed for multiwell plates in the anaerobic chamber [16–20, 32], with important efforts directed toward evaluating and optimizing medium formulation [17, 18, 20, 33–36], streamlining the workflow from feces to sample analysis [19, 37], and providing detailed technical procedures for cultivation [38].

Here, we aimed to contribute to the field by addressing remaining gaps in anaerobic high-throughput cultivation, including the need for (i) characterizing the influence of the gas phase on pH control and fecal microbiota growth and metabolism, (ii) designing a highly customizable medium preparation process to enable the testing of a wide range of factors and their combinations, and (iii) establishing conditions capable of maintaining the main characteristics of fecal inoculum from healthy donors while mitigating the bloom of *Enterobacteriaceae*, i.e. a taxonomic group associated with dysbiosis (e.g. irritable bowel syndrome, inflammatory bowel disease, obesity, colorectal cancer) [39] and prone to overgrowth during *ex vivo* cultivation [16, 18, 20, 21, 36, 40]. At the core of our approach is the use of a standardized basal medium that can be flexibly modified to test fecal microbiota responses to factors related to diet, drugs, and the host, individually or in combination. We highlighted the abiotic and biotic effects of the gas phase, buffer composition, and the cultivation process in a 96-deepwell plate within an anaerobic chamber (open gas-exchange system) compared to the well-established Hungate technique (closed tube-based system). Importantly, we evaluated the capacity of distinct mixtures of fibers and mucin to maintain the composition and activity of the gut microbiota from healthy adult donors while preventing *Enterobacteriaceae* overgrowth*.* Finally, we validated our protocol by showing similar *ex vivo* responses to known dietary fibers (resistant dextrin, soluble starch) and drugs (ciprofloxacin, 5-fluorouracil [5-FU]) as previously reported *in vivo.* A comprehensive protocol detailing the necessary steps and procedures for material preparation, high-throughput cultivation, and sample handling for analyses is provided in the Supplementary File 1.

## Materials and methods

### Growth media and anaerobic procedures

**Figure 1:**
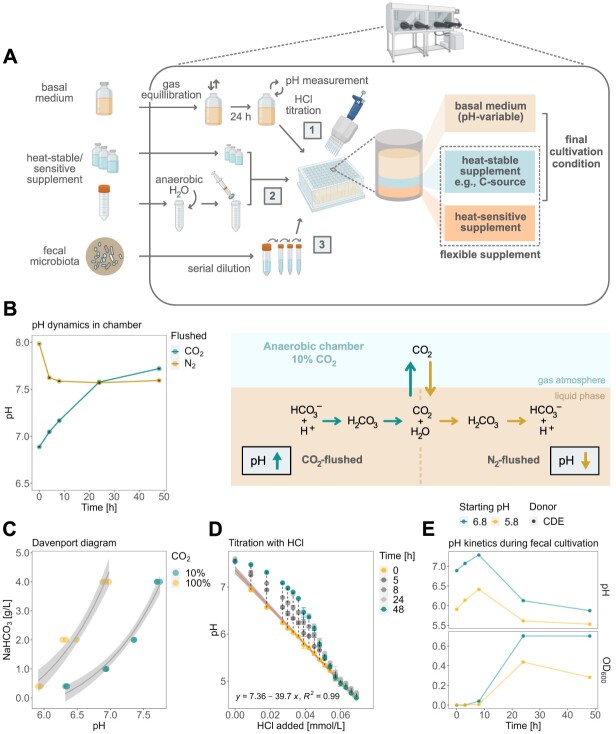
Overview of the high-throughput cultivation protocol and effect of the anaerobic chamber atmosphere on the medium’s chemistry and microbial physiology; (A) overview of the step-by-step cultivation protocol adapted for the anaerobic chamber setup; in a 96-deepwell plate, heat-stable/sensitive supplements (1) are mixed with HCl-titrated bYCFA (2) and inoculated with diluted feces (3); (B) characterization of pH dynamics of CO_2_ and N_2_-flushed bYCFA (uninoculated) in a 96-deepwell plate in an anaerobic chamber; (C) relationship between pH, aqueous NaHCO_3_ concentration, and gaseous CO_2_ levels (~10% in the anaerobic chamber, ~100% in gas-tight tube) in bYCFA after 48-h equilibration; (D) change of pH over time upon titration of bYCFA with HCl, with subsequent pH increase over 48 h; the resulting titration curve (at time 0 h) can be used to adjust the pH to different values; (E) pH and growth (OD_600_) during 48-h batch fecal cultivation in bYCFA (initial pH 6.8 and 5.8; containing 4 g/l NaHCO_3_ and 3 g/l resistant dextrin) titrated with HCl immediately before inoculation (donor CDE; 1% inoculation of 10^−4^ fecal dilution); data are presented as the mean and standard deviation of three technical replicates; all pH dynamic analyses were conducted in the 60 central experimental wells to circumvent the edge effect (Supplementary File 1, Step 4).

To cultivate fecal microbiota and pure cultures, we modified the yeast casitone fatty acid (YCFA) medium [41]. Specifically, we reduced nitrogen (N)-sources 10-fold, resulting in a basal minimal media, referred to as basal YCFA (bYCFA). bYCFA consisted of (L^−1^): 1 g amicase (Sigma-Aldrich Chemie GmbH, Buchs, Switzerland), 1.25 g yeast extract (Lesaffre, Marcq-en-Barœul, France), 0.5 g meat extract (Sigma-Aldrich), 150 ml mineral solution I (0.52 g K_2_HPO_4_), 150 ml mineral solution II (0.52 g KH_2_PO_4_, 0.9 g NaCl, 0.9 g (NH_4_)_2_SO_4_, 90 mg MgSO_4_, 90 mg CaCl_2_), 1 g L-cysteine HCl, and 4 g NaHCO_3_, 0.1 ml vitamin solution (10 μg biotin, 10 μg cobalamin, 30 μg 4-aminobenzoic acid, 50 μg folic acid and 150 μg pyridoxamine, Sigma-Aldrich), and 6.2 ml of volatile fatty acids solution (2.11 ml acetic acid (Sigma-Aldrich), 0.74 ml propionic acid (Sigma-Aldrich), 0.12 ml valeric acid (VWR International AG, Dietikon, Switzerland), 0.12 ml isovaleric acid (Sigma-Aldrich), and 0.12 ml isobutyric acid (Sigma-Aldrich) in 3.1 ml 5 M NaOH). The basal medium was prepared 1.33-fold concentrated since it was then complemented with supplements within the chamber (Fig. 1A; Table S1). Thus, all compounds except L-cysteine and NaHCO_3_ were dissolved in 75% of the final volume. The basal medium was adjusted to pH 7, boiled (10 min), and the remaining compounds were added after cooling to ~60 °C while constantly flushing with CO_2_ (or N_2_). After another 10 min, the medium was transferred into DURAN® Pressure flasks for plate-based cultivation (DWK Life Sciences GmbH, Wertheim, Germany) or Hungate tubes for tube-based cultivation (Belleco Glass, Vineland, NJ) and autoclaved.

To complement the basal medium, a range of heat-stable and heat-sensitive supplement solutions were prepared (Table S2). Heat-stable compounds were dissolved in dH_2_O, adjusted to pH 7, boiled for 10 min, cooled under N_2_, and autoclaved separately soon after. Heat-sensitive compounds were dissolved in anaerobic dH_2_O (except for omeprazole, which was dissolved in dimethyl sulfoxide (DMSO); 0.2% final concentration) in the anaerobic chamber, adjusted to pH 7, and filter sterilized.

The day before the experiment, the autoclaved basal medium was equilibrated with the gas phase of the anaerobic chamber (10% CO_2_, 5% H_2_, and 85% N_2_, Coy Laboratory Products Inc., Grass Lake, MI) under constant stirring, without the lid but covered with Breathe-Easier Sealing film (Diversified Biotech, Dedham, MA). On the day of the experiment, the pH was adjusted to 6.8 (if not indicated otherwise) by adding HCl 3 M based on a predetermined titration curve (Fig. 1E) and was mixed with the corresponding heat-stable/sensitive supplements (Tables S1 and S2).

A complete step-by-step description of the preparation of the bYCFA medium and heat-stable/sensitive supplement solutions for the high-throughput cultivation in 96-deepwell plates (Ritter GmbH, Schwabenmünchen, Germany) is presented in the Supplementary File 1.

### Fecal sample collection and *ex vivo* cultivation

Fourteen healthy donors (7 females and 7 males, ages ranging between 26 and 36) consented to provide one or more fresh fecal samples. Donors were excluded if they had taken antibiotics in the past 6 months. The sample collection was processed in an anonymized (using randomly generated three-letter codes) and non-interventional manner, and the ethics committee of ETH Zurich exempted this study from review.

Self-collected samples were provided in a plastic container containing an Oxoid™ AnaeroGen™ pouch (Thermo Fisher Diagnostics AG, Pratteln, Switzerland) to generate anaerobiosis during transport. Within 3 h, samples were relocated into the anaerobic chamber. Approximately 1 g of fecal matter was transferred using a plastic spoon into a 50 ml Falcon tube and homogenized with 9 ml of anaerobic phosphate buffer (pH 7.0). The resulting 10^−1^ dilution was then further diluted to 10^−4^ and inoculated (1%, v/v, in triplicates) into a plate (2 ml) or tube (8 ml) containing previously pH-adjusted basal medium complemented with respective supplements as listed in Table S1. After incubation for 48 h at 37 °C, cultures were transferred out of the chamber and centrifuged (5′500 rpm, 20 min and 4 °C). Cell pellets and supernatants were recovered and stored separately (−80 °C) until further processing (Supplementary File 1, Step 8).

Technical replicates exhibited small compositional differences (median Bray-Curtis distance 0.11) compared with the between-sample differences (median Bray–Curtis distance 0.72; Fig. S1). Therefore, when indicated, replicates were pooled before centrifugation by transferring 0.5 ml from each culture into a fresh plate (Supplementary File 1, Step 8) to reduce sample analysis time, cost, and storage space.

### Cultivation of pure intestinal strains

A panel of 10 human intestinal strains, representing diverse fiber and intermediate metabolite utilizers, were assessed for their growth and metabolic capabilities using the plate- and tube-based technique. The strains were acquired from the German Collection of Microorganisms and Cell Culture GmbH (DSMZ, Braunschweig, Germany) and PharmaBiome AG (Zürich, Switzerland; Table S3). They were stored at −80 °C in anaerobic glycerol stocks (25%, v/v) and reactivated by inoculating (1.25%, v/v) Hungate tubes containing bYCFA (pH 6.8) supplemented with the respective carbon sources (final concentrations of 3 g/l for fibers, 30 mM for glucose and intermediate metabolites; Table S3). Precultures were incubated for 24 h at 37 °C before experiments. Then, plates and tubes were filled with bYCFA, complemented with the corresponding C-sources, and inoculated (1% v/v). After incubation (48 h, 37 °C), 200 μl of cultures were transferred into a 96-well plate for OD_600_ measurement. Supernatants were recovered by centrifugation (4700 rpm, 20 min and 4 °C) for organic acids quantification.

### pH dynamics and bicarbonate buffering system in the anaerobic chamber

We characterized the relationship between pH, NaHCO_3_ concentration, and CO_2_ levels (0% or 100% in gas-tight tubes, compared to 10% in the anaerobic chamber) by measuring the pH of the basal medium containing different NaHCO_3_ concentrations (0.4, 1, 2, and 4 g/l). When required, pH dynamics were monitored over time. Measurements were performed in a 96-deepwell plate filled with 1.5 ml basal medium using a pH microelectrode (diameter 6 mm; Metrohm AG, Herisau, Switzerland) and a pH meter (type 913; Metrohm AG). A titration of bYCFA medium with HCl 3 M was performed and served as a calibration to support the pH adjustment of bYCFA in the chamber (Supplementary File 1, Step 6).

### Organic acids quantification

Short-chain fatty acids (SCFAs; acetate, propionate and butyrate) and intermediate metabolites (formate, lactate and succinate) were quantified by high-performance liquid chromatography equipped with a refractive index detector (HPLC-RI) as reported before [14]. All listed compound concentrations were corrected with blank values of the basal medium to estimate the net production of SCFA and intermediates. To quantify total metabolites, blank-corrected concentrations were summed, and metabolite fractions were calculated by dividing concentrations of respective metabolites by total metabolites.

### DNA extraction and 16S rRNA gene sequencing

Cell pellets were preprocessed using the Maxwell® HT 96 gDNA Blood Isolation Kit (Promega AG, Dübendorf, Switzerland) combined with a FastPrep homogenizer (FastPrep-24TM; MP Biomedicals, Illkirch-Graffenstaden, France), and followed by DNA purification using KingFisher Flex instrument (Thermo Fisher Scientific, Waltham, MA), according to the manufacturer’s instructions. The total DNA concentration after extraction was quantified using the Qubit® dsDNA HS Assay kit. Amplification and barcoding of the V4 hypervariable region of the 16S rRNA gene were performed using a polymerase chain reaction (PCR) with the barcoded primer 515F (5′-GTGCCAGCMGCCGCGGTAA-3′) and 806R (5′-GGACTACHVGGGTWTCTAAT-3′) [42, 43]. The pooled library was then sequenced using the amplifying primers (515F and 806R) and the Illumina MiSeq platform [44] at the Genetic Diversity Centre (ETH Zurich, Switzerland).

Sequencing reads were processed using the metabaRpipe R package (v0.9) [45]. In brief, reads were inferred with the DADA2 R package (v1.14.1) [46] with read filtering set to c(260, 250) and maximal error rates set to c(3, 4). Bimeras were removed, and taxonomy was assigned to amplicon sequence variants (ASVs) using the SILVA database (v138.1) [47, 48]. Reads were filtered with a per-sample abundance threshold of 0.1% [49] and then analyzed using the phyloseq package (v1.40.0) of the R software (v4.2.0) [50]. Alpha and beta-diversity analyses were done on rarefied samples (with an average rarefication depth of 2374 reads). Data meeting the criteria of homoscedasticity were evaluated with a permutational multivariate analysis using the adonis2 function (999 permutations) of the vegan package (v2.6–4). Differential abundance analysis was performed on non-rarified data using ALDEx2 [51]. Compositional taxa bar plots were generated using comp_barplot from the microViz package (v0.10.8) [52]. Using the aldex.clr function from the mia package (v1.5.17), the data were centered log(2)-ratio (clr) transformed using 1000 Dirichlet Monte-Carlo instances [53]. Significantly altered taxa were identified based on the *P*-value of Welch’s *t*-test, corrected according to the Benjamini–Hochberg Method.

### Data analysis and visualization

Data were analyzed using R software (v4.2.0) and visualized using the ggplot package (v3.3.6). Pearson correlation lines were plotted using ggpmisc (v0.5.1). Data were evaluated for normality using the Shapiro–Wilk Normality Test and when *P* > .05, the comparison of means was performed using a t-test. Otherwise, a Wilcoxon test using the rstatix package (v0.7.0) was used. *P*-values were corrected for multiple testing using the Holm–Bonferroni method. All codes used for data analysis in this study have been made publicly available and can be accessed at https://github.com/janinazuend/HTP.

## Results

### A modular system for medium preparation and pH control for high-throughput cultivation in the anaerobic chamber

To formulate a standardized high-throughput cultivation protocol, we opted for 96-deepwell plates, gas-permeable sealing membranes, and a standard anaerobic chamber. This setup enables simultaneous testing of multiple conditions, with 60 wells/plate allocated for experiments surrounded by water-filled wells to minimize evaporation and edge effects [54] (Supplementary File 1, Step 4), while wells at the edges of the plate can be used to allocate contamination controls. The working volume of 2 ml/well provides enough samples to conduct a range of analyses on the whole culture (e.g. OD_600_, pH), the cell-free supernatant (e.g. metabolites), and the cell pellet (e.g. DNA-based analyses) (Supplementary File 1, Step 8).

To provide flexibility for testing diverse culture conditions, we designed a modular procedure in which an anaerobic basal medium solution (containing N-sources, fatty acids, salts, buffers, trace elements, and heat-stable vitamins) is complemented within the chamber with a carbon (C)-source solution, and any other solution with specific components (heat stable/sensitive) to assess (Fig. 1A). As the basal medium, we selected bYCFA, which is a modified version of Yeast extract Casitone and Fatty Acids (YCFA) medium (C-depleted, 10-fold reduction N-sources), which has a long history of use in cultivating gut microbes and microbiota [41, 55]. The minimal nutrient availability of bYCFA enables the identification of supplement-specific responses.

Cultivation in anaerobic chambers poses diverse challenges, including maintaining strict anaerobiosis to prevent the proliferation of facultative anaerobes, such as *Enterobacteriaceae*. Our protocol highlights key practical steps for mitigating O_2_ contamination (Supplementary File 1, Step 2), with a particular focus on sustaining proper hydrogen levels (>2.5%) to achieve efficient O_2_ elimination and reproducible anaerobic microbial growth, as demonstrated in Fig. S2. Another challenge is pH control, which is influenced by the equilibrium between gaseous and dissolved CO_2_, with the latter forming carbonic acid (H_2_CO_3_) in an aqueous solution [56]. During the preparation of the basal medium, 100% CO_2_ or N_2_ is flushed to remove traces of O_2_ (Supplementary File 1, Step 1), resulting in dissolved CO_2_ levels that are out of equilibrium with the chamber atmosphere (here, 10% CO_2_). As a result, introducing CO_2_-flushed medium in the chamber led to a significant abiotic increase of pH (+0.16 ± 0.05 after 4 h and + 0.83 ± 0.03 after 48 h in bYCFA). In contrast, a rapid drop of pH was observed when introducing N_2_-flushed medium in the chamber (−0.36 ± 0.02 after 4 h in bYCFA, Fig. 1B). These changes in pH reflect the release of CO_2_ into the gas phase (CO_2_-flushed) or the solubilization of CO_2_ into the medium (N_2_-flushed) to equilibrate with the levels in the chamber’s atmosphere (Fig. 1B). The pH at equilibrium depended on the CO_2_ level in the atmosphere and on the concentration of NaHCO_3_ in the medium (Davenport diagram, Fig. 1C), which was set at 4 g/l in YCFA [41]. To establish a standard basal medium that can be flexibly adjusted to a range of pH values, we kept the original NaHCO_3_ concentration of 4 g/l and adjusted the pH of the medium (equilibrated in the chamber for 24 h) via HCl titration (Supplementary File 1, Step 6). This approach allowed rapid adjustment of the initial pH but also led to a slow increase in pH over time (Fig. 1D) as HCl titration shifts the bicarbonate buffer system toward CO_2_, which is gradually released again, resulting in alkalinization. The pH should thus be adjusted right before inoculation to maintain the initial pH in the desired range. To validate this approach, we titrated bYCFA with HCl (to pH 6.8 and pH 5.8) immediately before inoculation with fecal microbiota and incubated the cultures at 37 °C for 48 h (Fig. 1E). After an initial pH increase of +0.48 ± 0.07 (pH 6.8) and + 0.45 ± 0.02 (pH 5.8) after 8 h, the pH decreased and reached final values of 5.90 ± 0.04 (pH 6.8) and 5.51 ± 0.04 (pH 5.8) after 48 h (Fig. 1E). The degree of alkalinization resulting from the disrupted bicarbonate buffer was small compared to the acidification caused by microbial activity (i.e. organic acid production), and the pH remained within a range permitting the growth of common gut anaerobes [57]. Therefore, HCl-titration within the chamber was integrated into the protocol to rapidly set the initial pH to the desired value, thereby facilitating the testing of diverse pH. This step requires a pH meter in the chamber and a predetermined titration curve (Fig. 1D; Supplementary File 1, Step 6).

### Effect of the cultivation techniques on the microbial community and metabolism of fecal cultures

**Figure 2:**
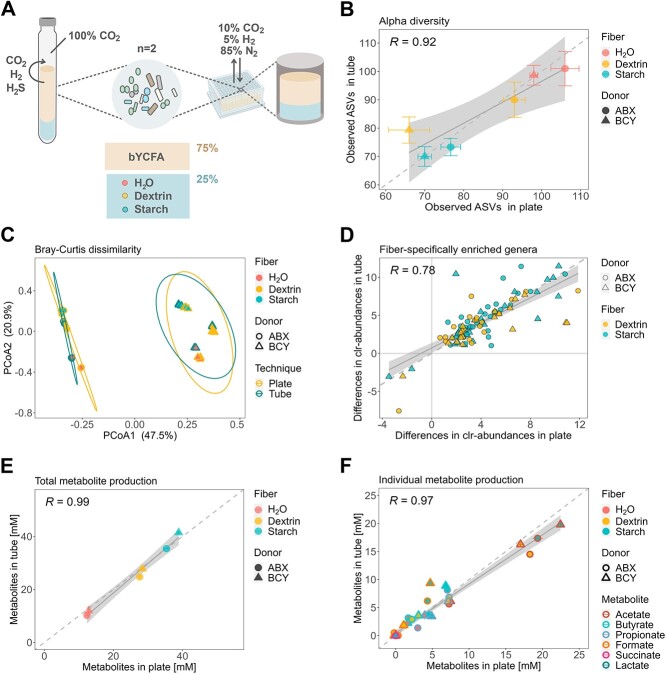
Impact of the cultivation technique (96-deepwell plates vs. gas-tight tubes) on the metabolism and composition of *ex vivo* cultures (donor ABX and BCY) cultivated in bYCFA for 48 h, 37 °C, in the presence of resistant dextrin (3 g/l), soluble starch (3 g/l), or H_2_O as control (technical triplicates); (A) experimental setup for comparing the plate- and tube-based techniques; all analyses were performed after 48h cultivation; (B) correlation of observed ASVs; points represent means, and bars represent standard deviations; (C) PCoA plot of Bray–Curtis distances with 95% confidence ellipses (calculated separately for each donor) for the two cultivation techniques; (D) taxa that are significantly different between each fiber (dextrin, starch) and H_2_O (*P* < .05); each data point corresponds to the median difference of the clr-abundance (genus-level) between plate and tube; (E) correlation of the total metabolite production; total metabolites were calculated by summing the medium-corrected concentrations of organic acids (SCFA and intermediates); (F) correlation of individual metabolites; points represent means of blank corrected concentrations and bars represent standard deviations.

Having established a modular procedure for preparing the medium and adjusting culture conditions, we next evaluated whether the plate-based cultivation system is equally suited to assess condition-specific microbial responses as the well-established and strict anaerobic Hungate technique. Indeed, differences might arise as a 96-deepwell plate can be regarded an open system, here exposed to an atmosphere of 10% CO_2_, 5% H_2_, and 85% N_2_. In turn, a Hungate tube functions as a closed system that is better protected from O_2_ contaminations, contains 100% CO_2_,and retains gases produced by microorganisms (e.g. CO_2_, H_2_, and H_2_S; Fig. 2A). Using both techniques, we cultured two fecal microbiota (48 h, 37 °C) in bYCFA containing two different C-sources (resistant dextrin, soluble starch, or H_2_O as control; Fig. 2A) that have previously shown to evoke strong microbial responses when using the Hungate technique [14]. As an indicator of microbial biomass [58], we observed similar or higher DNA concentrations in plates compared to tubes (Fig. S3A). The richness of fecal cultures (observed ASVs) strongly correlated between plates and tubes (R = 0.92, Fig. 2B); only cultures from donor BCY supplemented with dextrin exhibited lower richness in plates. Permutational multivariate analysis (Adonis2) of Bray–Curtis distances revealed that the cultivation technique significantly affected the community, albeit with a low effect size (F = 4.5, *P* ≤ .01; Fig. 2C), compared to the donor microbiota (F = 76.9, *P* < .001) and the supplemented C-source (F = 26.5, *P* ≤ .001). Despite slightly differing community structures, the taxa enriched in the two tested C-source conditions correlated between plates and tubes (R = 0.78, Fig. 2D). Notably, though not significant across all conditions and donors, plate cultivation is more prone to the proliferation of *Enterobacteriaceae* compared to tubes (Fig. S3B).

The concentrations of total metabolites (R = 0.99, Fig. 2E) and individual metabolites (R = 0.97, Fig. 2F) were similar between the two cultivation techniques. The main difference was a lower accumulation of the intermediate metabolite formate (*P* < .001, −3.5 ± 1.6) in plates as compared to tubes when starch was added (Fig. 2F). To identify whether the difference in metabolites produced might be attributed to changes in community composition or to variations in the metabolic outputs of individual strains exposed to the distinct gas atmosphere, we cultured a panel of pure gut bacteria using both techniques (Fig. S4A). The selected bacteria covered a range of nutritional preferences (e.g. primary degraders of complex fibers, utilizers of intermediate metabolites) and metabolic capabilities (e.g. acetate-, propionate-, and butyrate-producers). All bacteria grew well using both techniques, though six out of eight strains reached lower OD_600_ values after 48 h in plates compared to tubes (Fig. S4B). The cultivation technique influenced the carbohydrate metabolism of the tested strains, with all cultures exhibiting higher levels of end-metabolites (the SCFAs acetate, propionate and butyrate) in plates as compared to tubes (Fig. S4C). The two butyrate-producing species *Agathobacter rectalis* and *Eubacterium ramulus* yielded significantly lower levels of formate in plate compared to tubes (9.5 ± 2 vs. 12.5 ± 1 mM, *P* ≤ .05, and 1.1 ± 0.03 vs. 3.5 ± 0.1 mM, *P* ≤ .001, respectively; Fig. S4C). When grown in the chamber, the propionate-producing species *Phocaeicola dorei* showed higher conversion of succinate (1.8 ± 0.03 vs. 3.0 ± 0.1 mM, *P* ≤ .001) to propionate (5.7 ± 0.3 vs. 3.9 ± 0.1 mM, *P* ≤ .05; Fig. S4C) compared to tubes.

Collectively, the data indicate that cultivation in 96-deepwell plates results in similar community characteristics (alpha- and beta-diversity, DNA concentration) as the well-established Hungate technique. Fiber-specific taxonomic changes were highly comparable between the two protocols despite the higher susceptibility of plate cultures to *Enterobacteriaceae* proliferation. Furthermore, the open nature of the plate system and the composition of the gas used improved fermentability, favoring the conversion of C-sources to end-metabolites. Specific procedures for cultivating fecal microbiota (Supplementary File 1, Step 5.1) and pure cultures (Supplementary File 1, Step 5.2) are detailed in the high-throughput protocol.

### Formulation of a growth medium to mimic the microbial composition of healthy adult feces during *ex vivo* cultivation

**Figure 3:**
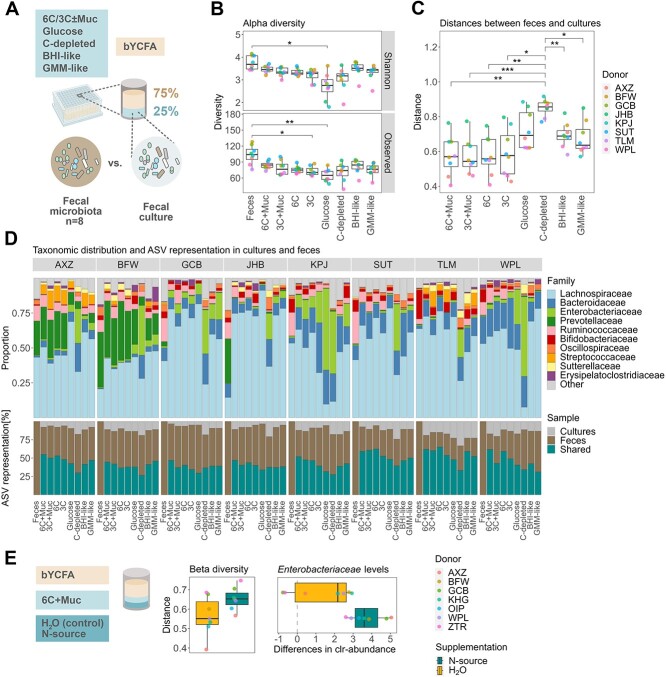
Formulation of a growth medium maintaining the microbial composition of healthy adult feces in *ex vivo* cultures during high-throughput cultivation (48 h, 37 °C) in bYCFA medium; (A) experimental setup for determining the optimal C-source mix to maintain the composition of the original feces in cultures; all analyses were performed after 48-h cultivation; (B) number of observed ASVs and Shannon-Index in feces and cultures with different C-sources; (C) Bray–Curtis distances between the feces and the respective culture; (D) visual representation of the community composition (top) and the shared ASVs between feces and cultures (bottom); taxonomic relative abundance values are provided in Supplementary File 2; (E) evaluation of the impact of additional N-source supplementation (amicase and yeast extract) on Bray-Curtis distances and on the difference in *Enterobacteriaceae* clr-abundance as compared to the feces; all analyses were performed on pooled samples from three independent replicates for each donor microbiota; a total of 11 distinct donors were used, i.e. AXZ, BFW, GCB, JHB, KPJ, SUT, TLM, WPL, KHG, OIP, and ZTR; multiple comparisons across all conditions were performed using a *t*-test and *P*-values were corrected using the Holm–Bonferroni method; statistically significant results are marked by stars, with ^*^ indicating *P* ≤ .05, ^**^P ≤ .01, and ^***^P ≤ .001.

Next, we sought to formulate an optimal mix of C-sources to complement bYCFA basal medium and maximize the compositional similarity between *ex vivo* cultures and feces from human adults. Addressing a previously identified challenge in plate-based systems, we also assessed whether we could minimize the commonly observed *ex vivo* overgrowth of *Enterobacteriaceae*, a family associated with dysbiosis [39]. By maintaining the main characteristics of the donor’s community in culture, we aimed to enhance the relevance of *ex vivo* testing in assessing the effect mediated by diet-, drug-, and host-related factors. We cultivated fecal samples from eight healthy adults and examined whether the bacterial composition of feces could be better maintained using complex mixtures of carbohydrates (3C and 6C; 3 g/l total) and mucin (±Muc), as compared to glucose and H_2_O (C-depleted control; Fig. 3A). The composition of 3C was designed to mirror the C-sources present in the M2GSC medium (33% starch, 33% cellobiose, and 33% glucose), which supports the growth of numerous gut microbes [55]. In comparison, 6C contained the carbohydrates found in the Macfarlane medium (15% starch, 15% pectin, 15% xylan, 8% arabinogalactan, 8% guar gum, and 38% inulin) [59], a nutrient-rich medium that closely mimics the gut chyme of healthy adults [59], and is commonly used in continuous intestinal *in vitro* models [60]. Finally, we incorporated two conditions designed to replicate the nutritional composition of media previously used for gut microbiota culturing, namely Brain Heart Infusion (BHI) medium [20, 61] and Gut Microbiota Medium (GMM) [20, 35, 61]. Since we showed that the bicarbonate concentration affects the pH within the chamber, and given that BHI and GMM inherently lack bicarbonate, we formulated BHI-like and GMM-like media to improve the comparability of results without introducing confounding factors related to pH. Specifically, the BHI-like and GMM-like media are composed of bYCFA, supplemented with the necessary C- and N-sources to match the composition of BHI and GMM (Table S2).

The fecal richness (observed ASVs with relative abundance >0.1%; median of 104 among donors) was best maintained in culture conditions containing both mucin and complex C-sources, with the 6C + Muc condition exhibiting the highest median of observed ASVs (84.5) together with BHI-like (84.5), followed by GMM-like (76.5), 3C + Muc (76.5), 6C (75.0), 3C (70.5), and glucose (65.0; Fig. 3B). A similar trend was observed when including microbial evenness (Shannon-Index; Fig. 3B). Though 74 ASVs were detected in the C-depleted controls, a significant portion of detected taxa potentially originated from resting or dead cells from the fecal inoculum, as limited growth (OD_600_) was observed after 48 h (Fig. S5A). When comparing the taxonomic composition of the fecal cultures to the original feces using Bray–Curtis distances (ranging from 0: all ASVs shared, to 1: no ASVs shared), the conditions containing complex C-sources demonstrated significantly higher similarity to the feces (6C + Muc: 0.57; 3C + Muc: 0.54; 6C: 0.56; and 3C: 0.58) than the C-depleted conditions (H_2_O: 0.86) and, although not significantly, than those with glucose (0.70), BHI- (0.69) and GMM-like supplements (0.64; Fig. 3C). In line, shared ASVs between feces and cultures were highest in 6C + Muc (53 ± 7%), followed by 6C (51 ± 11%), 3C + Muc (48 ± 9%), 3C (44 ± 7%), BHI-like (43 ± 7%), GMM-like (43 ± 7%), and lowest with glucose (39 ± 7%) or in the absence of a C-source (33 ± 5%; Fig. 3D). At the family level, the main fecal taxa were represented in cultures containing complex C-sources (Fig. 3D), except for donor JHB, for which a relative loss of *Prevotellaceae* abundance was observed *ex vivo* (0.5 ± 0.4% among all complex C-source cultures vs. 31.8% in feces; Fig. 3D). Notably, adding mucin and complex C-sources limited the relative growth of *Enterobacteriaceae,* with the smallest increase in clr-abundance between cultures and feces observed with 6C + Muc (2^1.2^-fold increase) and the largest in the presence of glucose (2^3.2^-fold increase) and H_2_O (2^4.3^-fold increase; Fig. S5B). Overall, among all cultures containing 6C + Muc, the maximum relative abundance of *Enterobacteriaceae* detected after 48 h was 9% (Donor KPJ; Fig. 3D).

When evaluating the metabolic outputs of fecal cultures, a significant increase in metabolite production was observed in the presence of complex C-sources (Fig. S5C). However, substantial amounts of formate accumulated (Fig. S5D), indicating incomplete fermentation. Conversely, BHI-like conditions resulted in more metabolites production compared to the 6C + Muc condition, with lower relative concentrations of formate (1.3 ± 1.5% vs. 8.5 ± 3%; Fig. S5D). Thus, we assessed whether the addition of N-source in the 6C + Muc condition could promote end-metabolite production [62]. We cultivated fecal samples from seven healthy adults in bYCFA with 6C + Muc and supplemented an additional 7.2 g/l amicase and 1 g/l yeast extract (Fig. 3E). With additional N-sources, a reduction of formate levels after 48 h (from 14.4 ± 3.8% to 1.3 ± 2.6%) and a representative SCFA ratio [63] was observed, with 61.5 ± 5.1% acetate, 18.9 ± 1.9% butyrate, and 16.4 ± 5.4% propionate among all donors (Fig. S6A). However, N-source addition did not help maintain the composition of the original feces and resulted in a slight reduction of richness (74 observed ASVs with extra N-sources and 81 ASVs in control; Fig. S6B) and increased Bray–Curtis distances between cultures and feces (extra N-source: 0.65; control: 0.55; Fig. 3E). Notably, the clr-abundance of *Enterobacteriaceae* was enhanced by additional N-sources (Fig. 3E). Taken together, we recommend complementing bYCFA with 6C + Muc without additional N-sources to better retain the compositional characteristics from adult feces and limit *Enterobacteriaceae* bloom during cultivation (Supplementary File 1).

Lastly, we evaluated the stability of *ex vivo* culture compositions in bYCFA with 6C + Muc over multiple passages to provide a potential setup for studying microbial community recovery patterns after perturbations [16]. Two fecal cultures were re-inoculated (1% v/v) into fresh medium over three successive passages (P1, P2, P3) at two different intervals (24 or 48 h; Fig. S7A). Repeated inoculation maintained the cultures’ richness (Fig. S7B) but increased the dissimilarity between the cultured communities and the original fecal samples (Bray–Curtis distances; Fig. S7B). These findings indicate that although re-inoculation shifts the community structure away from the original fecal composition, this simple approach could serve for studying the recovery dynamics of gut microbes after perturbations (e.g. applied in P1, with recovery evaluated in P2 onwards), provided that appropriate controls are implemented to account for community shifts.

### Treating *ex vivo* cultures with dietary fibers or drugs results in physiologically relevant microbial responses

**Figure 4:**
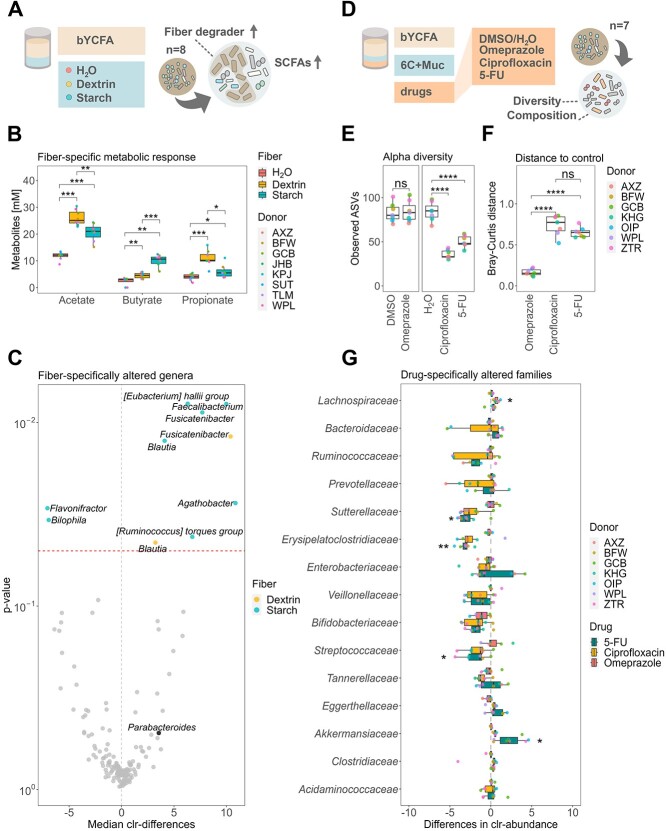
Treatment with dietary fibers (donor *n* = 8) or drugs (donor *n* = 7) elicits physiologically relevant changes in the *ex vivo* cultures’ metabolism and community composition (bYCFA, 48 h, 37 °C); (A) experimental setup to test the fiber-specific response of fecal cultures. All analyses were performed after 48-h cultivation; (B) SCFA production by fecal cultures when bYCFA was supplemented with resistant dextrin (3 g/l), soluble starch (3 g/l), or no C-source (H_2_O as control); (C) median clr-differences of taxa significantly differed between each fiber (dextrin, starch) and H_2_O; genera significantly different (*P* ≤ .05) are labeled and located above the dotted red line; (D) experimental setup to test the effect of drugs (i.e. omeprazole, ciprofloxacin and 5-FU) as compared to the vehicle control (DMSO or H_2_O) in bYCFA supplemented with 6C + Muc; all analyses were performed after 48h cultivation; (E) number of observed ASVs as compared to the vehicle control; (F) Bray–Curtis distances between controls and the drug-treated cultures; (G) Clr-differences of the 15 most abundant families; significantly altered families, as compared to the control, are marked by stars; all the analyses were performed on pooled samples from incubations for each donor microbiota; multiple comparisons across all conditions were performed using a *t*-test, and *P*-values were corrected using the Holm–Bonferroni method; statistically significant results are marked by stars, with ^*^ indicating *P* ≤ .05, ^**^P ≤ .01, ^***^P ≤ .001, and ns, non-significant.

Finally, we aimed to validate our *ex vivo* high-throughput cultivation protocol by testing whether supplementation with specific dietary fibers (resistant dextrin and soluble starch; Fig. 4A) or drugs (omeprazole, ciprofloxacin, and 5-FU; Fig. 4D) could induce microbial changes that are consistent with previous *in vivo* findings.

We chose resistant dextrin (3 g/l) and soluble starch (3 g/l) as dietary fibers due to their well-known ability to modulate the gut microbiota and mediate propiogenic [64] and butyrogenic [65–67] effects, respectively. Using feces from eight healthy donors, we performed enrichment experiments in bYCFA containing each fiber as the sole C-source. Among all donors, we found significantly higher levels of propionate after 48 h in the presence of dextrin (10.9 ± 2.9 mM) compared to starch (6.0 ± 2.4 mM; *P* ≤ .05) or the control without C-sources (3.9 ± 1.2 mM; *P* ≤ .001). Conversely, starch supplementation led to significantly higher levels of butyrate (10.1 ± 2.1 mM) compared to dextrin (4.5 ± 1.1 mM; *P* ≤ .001) or the control (2.4 ± 1.5 mM; *P* ≤ .01; Fig. 4B). At the taxonomic level, resistant dextrin increased the relative abundance of *Fusicatenibacter* (*P* ≤ .05) and *Blautia* (*P* > .05) among all donors (Fig. 4C), consistent with previous *ex vivo* and *in vivo* studies [14, 68, 69]. Human trials also identified a dextrin-specific increase of *Parabacteroides* [14, 70], which was increased in our *ex vivo* cultures (2^3.5^-fold increase), though the effect was not significant. Starch supplementation most prominently enriched *Faecalibacterium* (*P* ≤ .01) and *Agathobacter* (*P* ≤ .05; formerly *Eubacterium,*  Fig. 4C), which were also promoted in previous human trials [25, 66, 67, 71]. Relative abundances of *Flavonifractor* (*P* < .05) and *Bilophila* were reduced with starch (*P* < .05; Fig. 4C).

Many commonly used medications have been shown to disrupt gut microbial communities [6, 72]. Conversely, microbial activities can alter drug efficacy and toxicity [73–75]. To test the direct interaction between the gut microbiota and drugs, we exposed the feces from seven individuals (in bYCFA with 6C + Muc) to a single dose of 11 μM omeprazole (proton-pump inhibitor), 50 μM ciprofloxacin (antibiotic), or 50 μM 5-FU (cytostatic), representing the respective expected intestinal concentrations [16, 76, 77]. Compared to the vehicle control cultures, treatment with ciprofloxacin and 5-FU negatively impacted the microbial communities’ diversity (Fig. 4E) and structure (Fig. 4F). Ciprofloxacin led to a relative increase in *Lachnospiraceae* (*P* ≤ .05) (Fig. 4G). The reduction in community diversity and persistence of *Lachnospiraceae* agrees with previous findings in mice [16] and ciprofloxacin-treated patients [78]. Treatment with 5-FU inhibited *Sutterellaceae* (*P* ≤ .05) and *Erysipelatoclostridiaceae* (*P* ≤ .01) and promoted the relative abundance of *Akkermansiaceae* (*P* ≤ .05) (Fig. 4G). Several mice studies have demonstrated the reduction in diversity and the drastic bloom of *Akkermansiaceae* in response to 5-FU [79–81]. In contrast, we observed no significant effect of omeprazole on fecal cultures at the tested concentration (Fig. 4E and F). This common proton pump inhibitor has been shown to decrease the richness and enrich the abundance of oral bacteria (e.g. *Streptococcaceae*) in the gut [82]. However, it was proposed that the effect of omeprazole on the gut microbiota might not be directly caused by the molecule but rather by an elevation of gastric pH, disrupting the barrier between the upper and lower gastrointestinal tract [82]. This hypothesis aligns with our *ex vivo* findings. It is worth noting that omeprazole was dissolved in DMSO at a final concentration of 0.2% DMSO in fecal culture. At this concentration, DMSO did not affect the communities’ diversity and structure (Fig. S8), indicating its suitability as a solvent for testing compounds with low solubility in an aqueous phase.

## Discussion

A well-controlled protocol for cultivating stool-derived microbial communities in the anaerobic chamber has the potential to advance our understanding of the causal interactions between gut microbes, diet, drugs, and host physiology [83]. Such a protocol should possess a high-throughput capacity to effectively capture the complex and individualistic nature of the gut microbiota, with heterogenous compositions among individuals [24–27], variations in activities at the strain level [28–31], and the influence of environmental factors on ecological and physiological dynamics [6, 10]. An increasing number of studies have reported the development of anaerobic high-throughput systems for cultivating stool-derived gut microbiota to generate personalized culture collections of gut microbes [84, 85], map the ability of the human gut microbiota to metabolize small molecule drugs [18, 75], or evaluate the response of fecal communities to specific stimuli [16, 17]. These studies have highlighted the promise of high-throughput *ex vivo* modeling by demonstrating a strong correlation between *ex vivo* and *in vivo* gut microbiota responses [16, 17]. Here, we addressed some of the practical, technical, and biological gaps in fecal cultivation that we consider essential to create a widely applicable, reproducible and accessible protocol, and advance the field of gut microbiota research.

First, we conducted an in-depth characterization of potential confounding factors associated with anaerobic chamber cultivation by comparing plate-based cultivation with the well-established Hungate technique. Plate cultures are more susceptible to O_2_ contamination than Hungate tube cultures, with a higher risk of disrupting the growth of commensals (e.g. *Faecalibacterium prausnitzii*, Figs S2) and promoting the commonly observed bloom of facultative anaerobes (e.g. *Enterobacteriaceae*, Fig. S3B). To minimize the risk of O_2_ contamination, we strongly recommend adhering to the manufacturer’s recommendations for proper chamber preparation by maintaining high H_2_ levels and regenerating the palladium catalysts (Supplementary File 1, Step 2). Although it is theoretically possible to generate anaerobiosis by prereducing a sterile aerobic medium inside the chamber for several days before the experiment [16–20], we recommend an additional step of medium boiling and flushing (CO_2_ or N_2_) prior to sterilization (Supplementary File 1, Step 1), as practiced in the Hungate technique [13]. Despite the increased relative *Enterobacteriaceae* abundances in plates compared to tubes, both protocols resulted in strong and comparable substrate-specific responses, indicating that both methods are equally suited for assessing supplement-specific responses.

Second, we highlighted that the equilibrium between gaseous and dissolved CO_2_ and the concentration of NaHCO_3_ in the solution influenced the pH of the medium in the chamber (Fig. 1B–E). The bicarbonate buffer is more challenging to control in open systems (e.g. 96-deepwell plates) where CO_2_ can escape. Still, the bicarbonate buffer optimally maintains pH in the range of the human adult colon from 5.7 (proximal) to 6.7 (distal) [86] when potential pH shifts are accounted for, e.g. by performing pH adjustment with HCl immediately before inoculation (Supplementary File 1, Step 6). Note that bYCFA basal medium also contains a phosphate buffer to maintain the pH between 6.4 and 7.4.

Third, we identified a few metabolic differences between cultivation in plates and tubes. The typically lower CO_2_ concentration in the chamber (~10%) compared to the concentration in tubes (~100%) mediated an increase of succinate to propionate conversion in pure cultures of *P. dorei* (Fig. S4C) [87, 88]. Moreover, the plate-based system allows microbially-produced gases to be released. H_2_, for instance, is a common growth-limiting factor that accumulates in tube cultures during fermentation [89] and promotes the accumulation of intermediate metabolites [90]. Our data support this finding, as tube cultures exhibited a higher accumulation of intermediate products (i.e. formate) than plate cultivation (Fig. 2C and Fig. S4C). Overall, the typical gas composition in the anaerobic chamber (i.e. 5% H_2_, 10% CO_2_, and 85% N_2_) is close to the physiological conditions reported *in vivo* for H_2_ and CO_2_, with concentrations of 2.9 ± 0.7% and 9.9 ± 1.6%, respectively [91].

Fourth, we convey our findings in a simple and comprehensive step-by-step protocol (Supplementary File 1), which includes a reproducible and modular medium preparation process. The basal medium bYCFA is heat-stable (sterilization via autoclave) and contains a limited amount of undefined ingredients to increase batch-to-batch reproducibility. Furthermore, it can be flexibly complemented with C-sources or any other molecules to test, and the pH can be easily adjusted, allowing for testing of a wide range of culture conditions with minimal medium preparation effort. Here, we showcased two examples of experiments by using the presented protocol. We first complemented bYCFA with defined fibers to identify taxa associated with functional niches (Fig. 4A–C). The results corroborated with a previous tube-based enrichment study, with the validity of this approach being further supported by a parallel nutritional intervention study [14]. We then tested the microbial responses to antibiotic and non-antibiotic drugs in fecal cultures (Fig. 4D–G), using an optimized mix of C-sources to maximize compositional similarity to feces (6C + Muc; Fig. 3B–D). This setup could be extended to study the effects of other xenobiotics (e.g. pollutants), dietary compounds (e.g. N-sources, vitamins), host-derived factors (e.g. pH, redox potential, oxidative stress, bile salts, antimicrobial peptides), or their combinations. Importantly, the medium composition containing 6C + Muc surpassed other commonly used media for fecal microbiota *ex vivo* cultivation, such as BHI-like and GMM-like, especially in maintaining the gut microbiota community structure (Fig. 3C and D). By complementing bYCFA with a mix of mucin and complex carbohydrates (6C + Muc), we were able to mitigate the blooming of *Enterobacteriaceae* (Fig. S5B). A previous study suggested that the *ex vivo* overgrowth of *Enterobacteriaceae* in the starch-containing medium, modified Gifu Anaerobic Medium, could be prevented by mixing it with the Bryant and Burkey medium [18, 20]. The latter contains acetate, a known inhibitor of *Enterobacteriaceae* taxa, such as *Escherichia coli* [92]. Another study highlighted the importance of mucin as a growth factor of *Firmicutes* [35]. Based on these previous findings and our data, it is tempting to speculate that combining mucin and complex carbohydrates in the 6C + Muc solution may foster the proliferation of commensals and, at the same time, organic acids in bYCFA may serve as growth inhibitors for *Enterobacteriaceae*.

Altogether, this protocol encompasses key features like flexibility (e.g. testing of various molecules and conditions alone or in combination; complex communities or pure cultures), reproducibility (e.g. reduced media complexity; preparation of stable stock solutions), and simplicity (e.g. minimized hands-on time; easy pH adjustment; reduced evaporation rate). It is important to recognize that common limitations of batch cultivation setups (e.g. limited cultivation time, nutrient depletion, acidification, accumulation of inhibitory metabolites) are inherent to the system presented [21]. Therefore, the applicability of the protocol must be carefully evaluated based on the research question and results have to be interpreted accordingly. Although batch cultivation models are valuable screening tools for growth capacities, metabolic potentials, and inter-individual variations, more complex *in vitro* models (i.e. continuous culture) are undoubtedly superior for in-depth studies of ecological and evolutionary dynamics, as they provide accurate readouts with stable conditions and continuous sampling. In addition, continuous fermentations are optimal for validating mechanism hypotheses generated with batch cultivation setups.

In conclusion, our high-throughput cultivation protocol is a valuable tool for fundamental and applied gut microbiota research. It enables rapid and reproducible characterization of diet-, host-, or drug-specific effects, microbe–microbe interactions [93], strain-specific [28, 31], and donor-specific [27] responses and activities. The protocol can also be applied to cost-efficient screening of drug metabolism by the gut microbiota [75, 76], preselection of treatments or dosage prior to costly animal or human trials [94], development of personalized microbiota-targeted nutritional interventions [95], identification of responders and non-responders to drugs and prebiotics [27], and for discovery and investigation of novel live biotherapeutics [96].

## Supplementary Material

Supplementary_Figures_Tables_Zund_et_al_ycae035

Supplementary_file_1_Zund_et_al_ycae035

Supplementary_file_2_Zund_et_al_ycae035

## Data Availability

All data reported in this paper are deposited in the ENA repository, with the accession number PRJEB68331, https://www.ebi.ac.uk/ena/browser/view/PRJEB68331.
