# Supplementary material for: A flexible high-throughput cultivation protocol to assess the response of individuals’ gut microbiota to diet-, drug-, and host-related factors
Source: ISME Commun. 2024 Mar 12;4(1):ycae035. doi: 10.1093/ismeco/ycae035 (PMC10982853; doi:10.1093/ismeco/ycae035)
Supplement: Supplementary_Figures_Tables_Zund_et_al_ycae035 [file supplementary_figures_tables_zund_et_al_ycae035.docx]

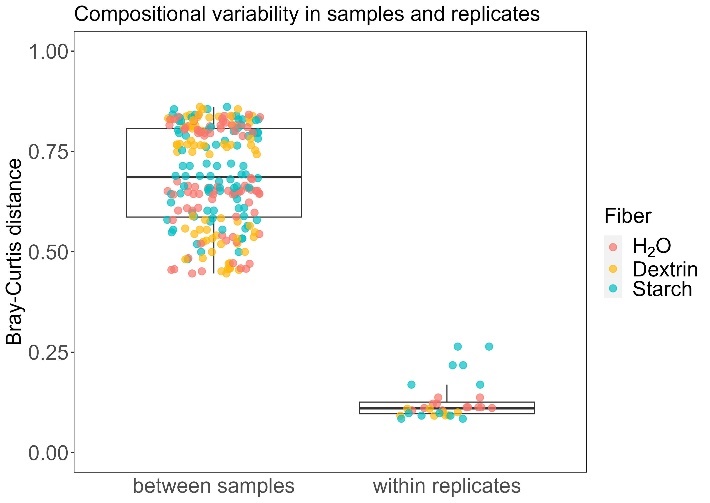


**Figure S1: Bray-Curtis distance between samples and within technical replicates of plate-cultured fecal communities (in bYCFA for 48 h, 37°C) in the presence of resistant dextrin (3 g/L), soluble starch (3 g/L) or H_2_O as control (technical triplicates).**

The displayed samples originate from the experiment comparing the two cultivation techniques (96-deepwell plates vs. gas-tight tubes). *Ex vivo* cultures (donor ABX and BCY) were cultivated in bYCFA for 48 h, 37°C, in the presence of resistant dextrin (3 g/L), soluble starch (3 g/L) or H_2_O as control using plates. Distances between samples include distances calculated between cultures under different cultivation conditions or originating from different donor microbiota, while the within replicate distances describe the distance between samples under the same conditions and from the same donor.


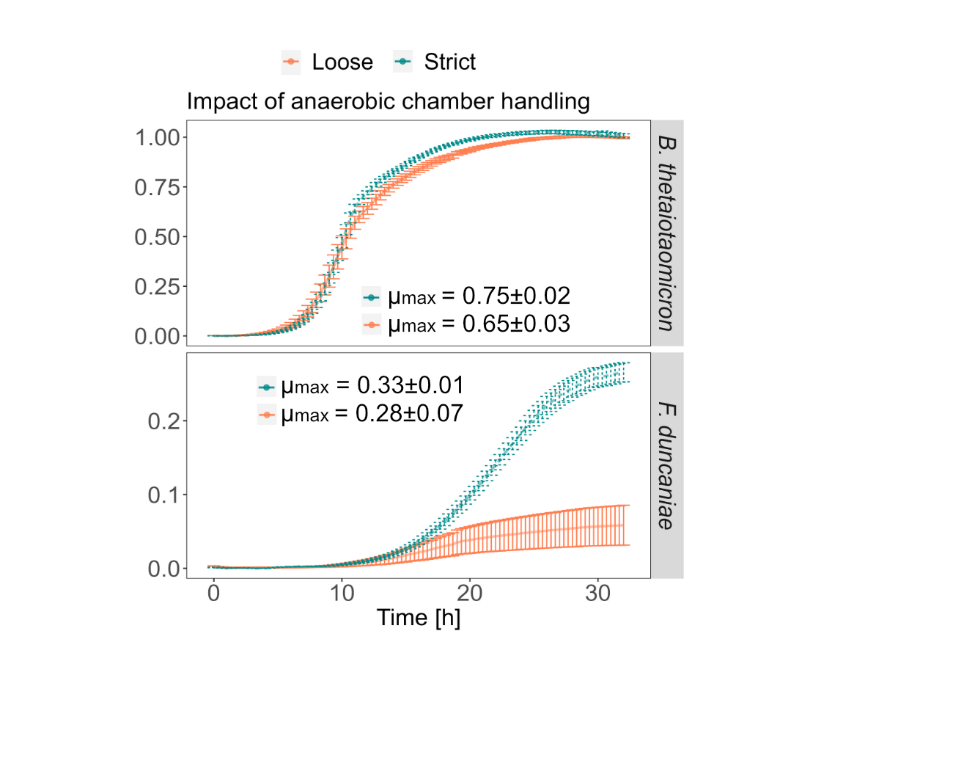


**Figure S2: The effect of chamber handling on growth kinetics (OD_600_) of *Bacteroides thetaiotaomicron* DSM 2079 and *Faecalibacterium duncaniae* DSM 17677 in bYCFA (30 mM glucose, pH 6.5, 37°C, 32 h).**

Overnight cultures (diluted to OD_600_ ∼0.2) were inoculated (1% v/v) into 200 µL bYCFA (30 mM glucose, pH 6.5) in a 96-wellplate and incubated in an anaerobic chamber that was handled “strictly” or “loosely”. A “strictly” handled chamber (**Supplementary file 1, Step 2**) consisted of rejuvenated palladium catalysts and a gas atmosphere replaced with fresh gas mix (10% CO_2_, 5% H_2_, and 85% N_2_), resulting in 0-20 ppm O_2_ and >2.5% H_2_ (monitored with an Anaerobic Monitor CAM-12, Coy Laboratory Products Inc.). To demonstrate the effect of a “loosely” handled chamber, non-rejuvenated palladium catalysts were introduced into the chamber, and gas mix was replaced with N_2_ until the H_2_ level was less than 1.5%. The maximum specific growth rate (μmax) was calculated according to first-order growth kinetics. μmax of *B. thetaiotaomicron* was significantly higher in the strict compared to the loose regime (0.75±0.02 vs. 0.65±0.03 h-1; p<0.05). Similarly, the maximal OD_600_ for *F. duncaniae* was significantly higher in the strict compared to the loose regime (0.27±0.02 vs. 0.06±0.04; p<0.01) Normality assumption was verified using the Shapiro-Wilk normality test, and significance was determined using an unpaired T-test. The significance level was set to p <0.05. Each condition was tested in biological triplicates.

**
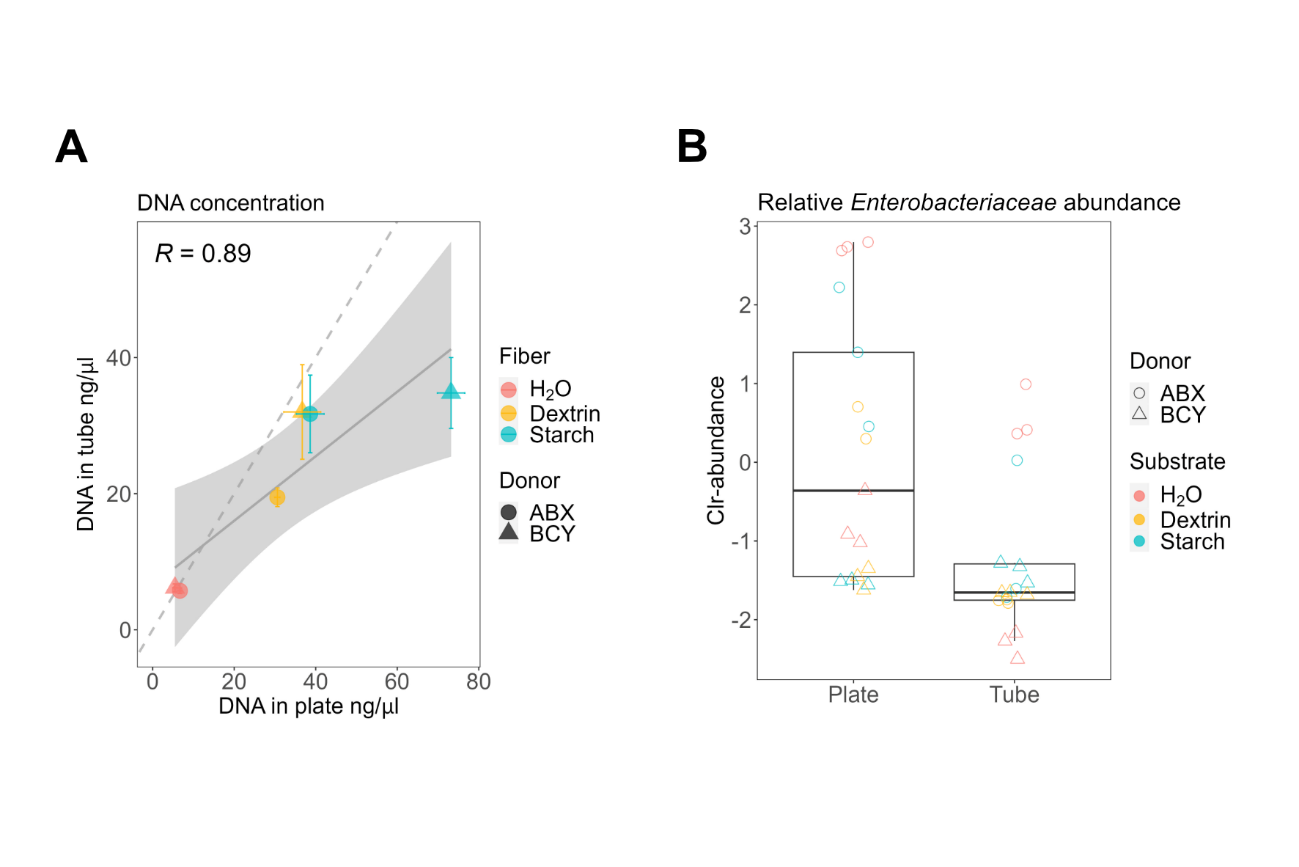
**

**Figure S3: Impact of the cultivation technique (96-deepwell plates vs. gas-tight tubes) on overall biomass (DNA concentration) and *Enterobacteriaceae* levels in ex vivo cultures (donor ABX and BCY).**

Fecal cultures were grown in bYCFA for 48 h, 37°C, in the presence of resistant dextrin (3 g/L), soluble starch (3 g/L) or H_2_O as control (technical triplicates). Each condition was tested in triplicates. **A)** DNA concentrations measured from 1 mL cell pellets extract, and analyzed using Pearson correlation. Points represent means and bars represent standard deviations. **B)** Clr-abundances of the *Enterobacteriaceae* family in plate compared to tube. Points represent individual replicates.

**
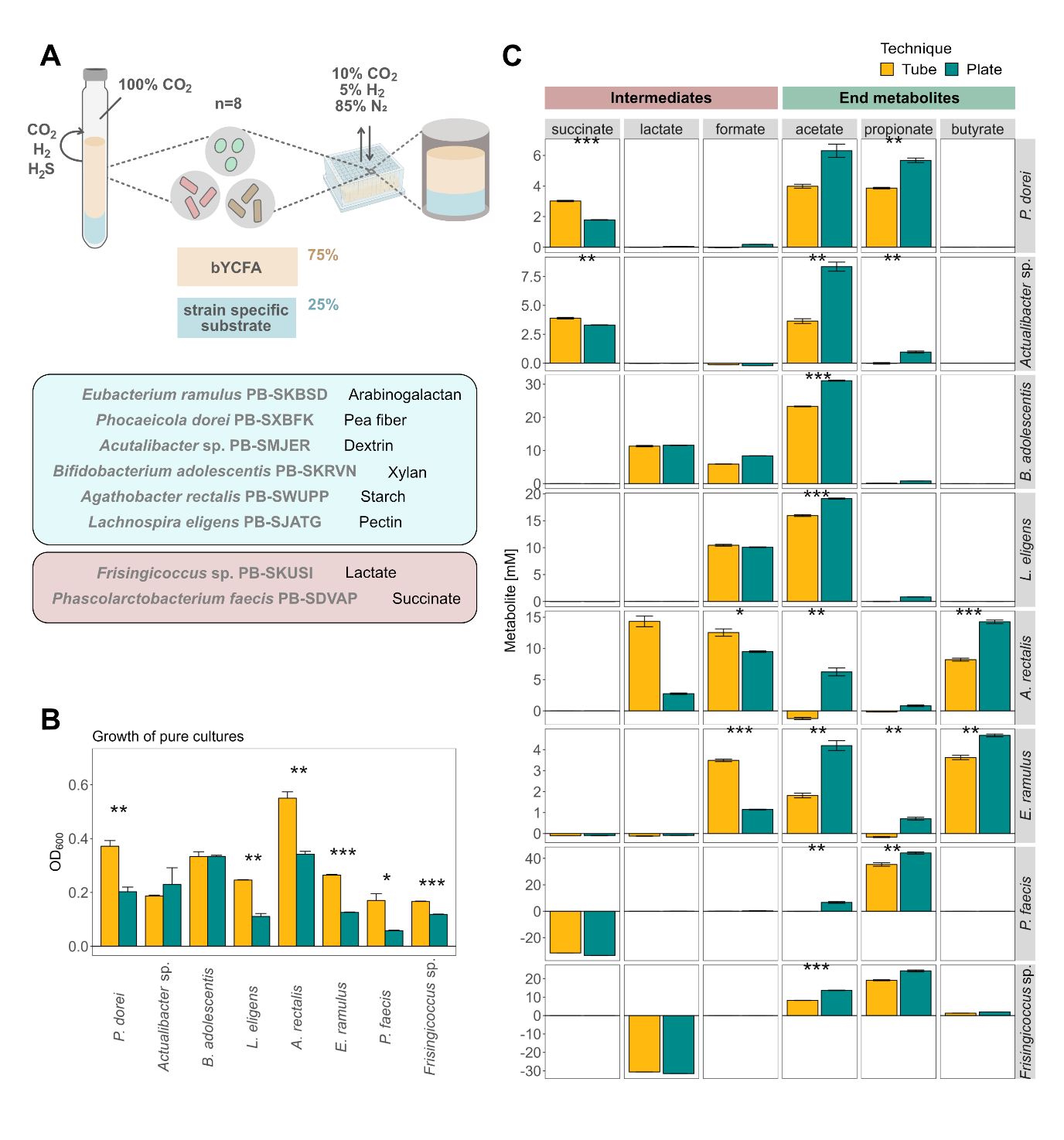
**

**Figure S4: Impact of the cultivation technique (96-deepwell plates vs. gas-tight tubes) on the physiology of pure bacterial strains cultivated in bYCFA for 48 h, 37°C, in the presence of different carbon sources (8 strains; biological triplicates).**

**A)** Experimental setup for comparing the high-throughput protocol with the Hungate technique using pure cultures and their preferred substrate as listed. All analyses were performed after 48 h cultivation. **B)** Growth of eight intestinal strains. Bars represent mean endpoint OD_600_ values. **C)** Individual metabolite production. Bars represent mean endpoint concentrations.

bYCFA was supplemented with strain-specific substrates (**Table S1**): *E. ramulus* with arabinogalactan, *P. dorei* with pea fiber, *P. faecis* with succinate, *Acutalibacter* sp. with resistant dextrin, *B. adolescentis* with xylan, *A. rectalis* with soluble starch, *Frisingicoccus* sp. with DL-lactate, and *L. eligens* with pectin. Each condition was tested in biological triplicates. Significance was assessed using a t-test with * indicating p≤0.05, ** p≤0.01 and *** p≤ 0.001.


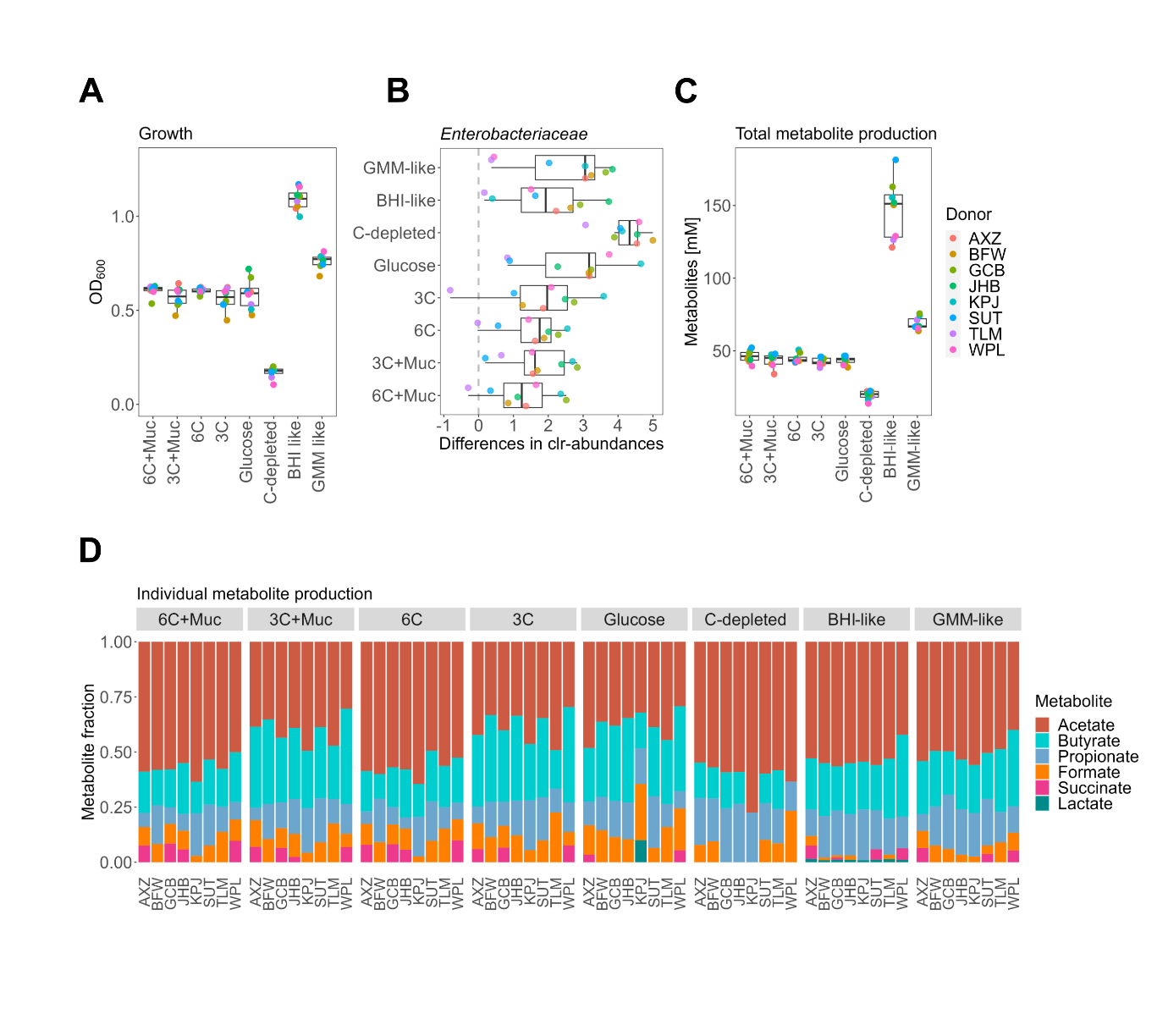


**Figure S5: Characteristics of *ex vivo* cultures (in bYCFA, 48 h, 37°C, donors n=8) grown in the presence of different C-sources (6C+Muc, 3C+Muc, 6C, 3C, glucose, BHI-like and GMM-like) or in control conditions (C-depleted).**

All analyses were performed after 48 h cultivation. **A)** Growth (OD_600_) of ex vivo cultures (200 µL sample; path length ~5 mm). **B)** Median difference of clr-abundance of *Enterobacteriaceae* in cultures compared to feces. **C)** Total metabolites were calculated by summing the medium-corrected concentrations of organic acids (SCFA and intermediates) in the supernatant of cultures. **D**) Relative metabolite production was calculated by dividing the concentration of each metabolite by the total amount of metabolites produced.


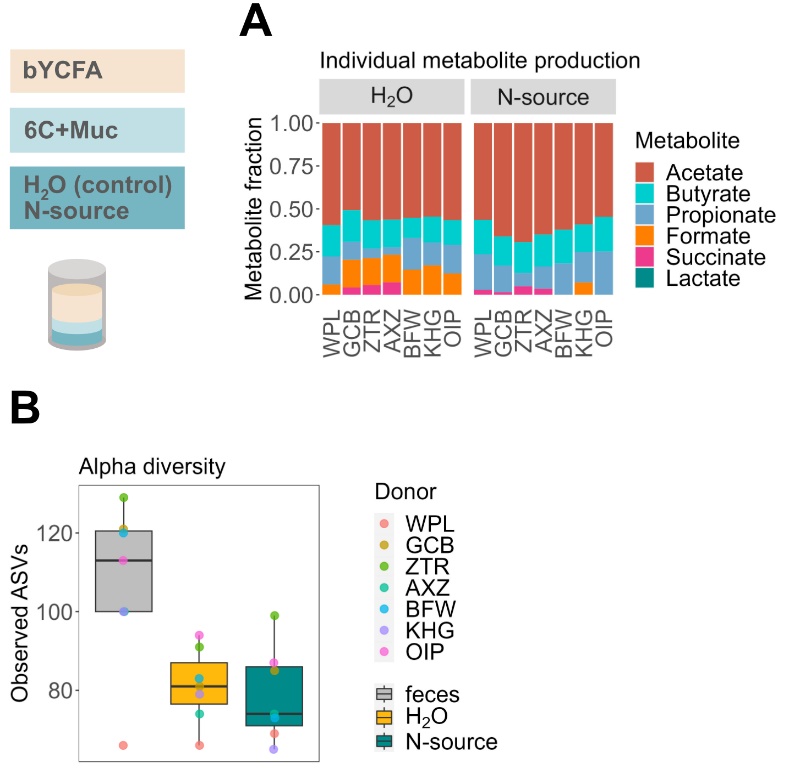


**Figure S6: Characteristics of *ex vivo* cultures grown in bYCFA with 6C+Muc (48 h, 37°C, donors n=7) supplemented with additional N-source (amicase and yeast extract) or control (H_2_O) conditions**

**A)** Relative metabolite production was calculated by dividing the concentration of each metabolite by the total amount of metabolites produced. **B)** Number of observed ASVs in feces and cultures.

All analyses were performed after 48 h on pooled samples from three technical replicates for each donor microbiota.


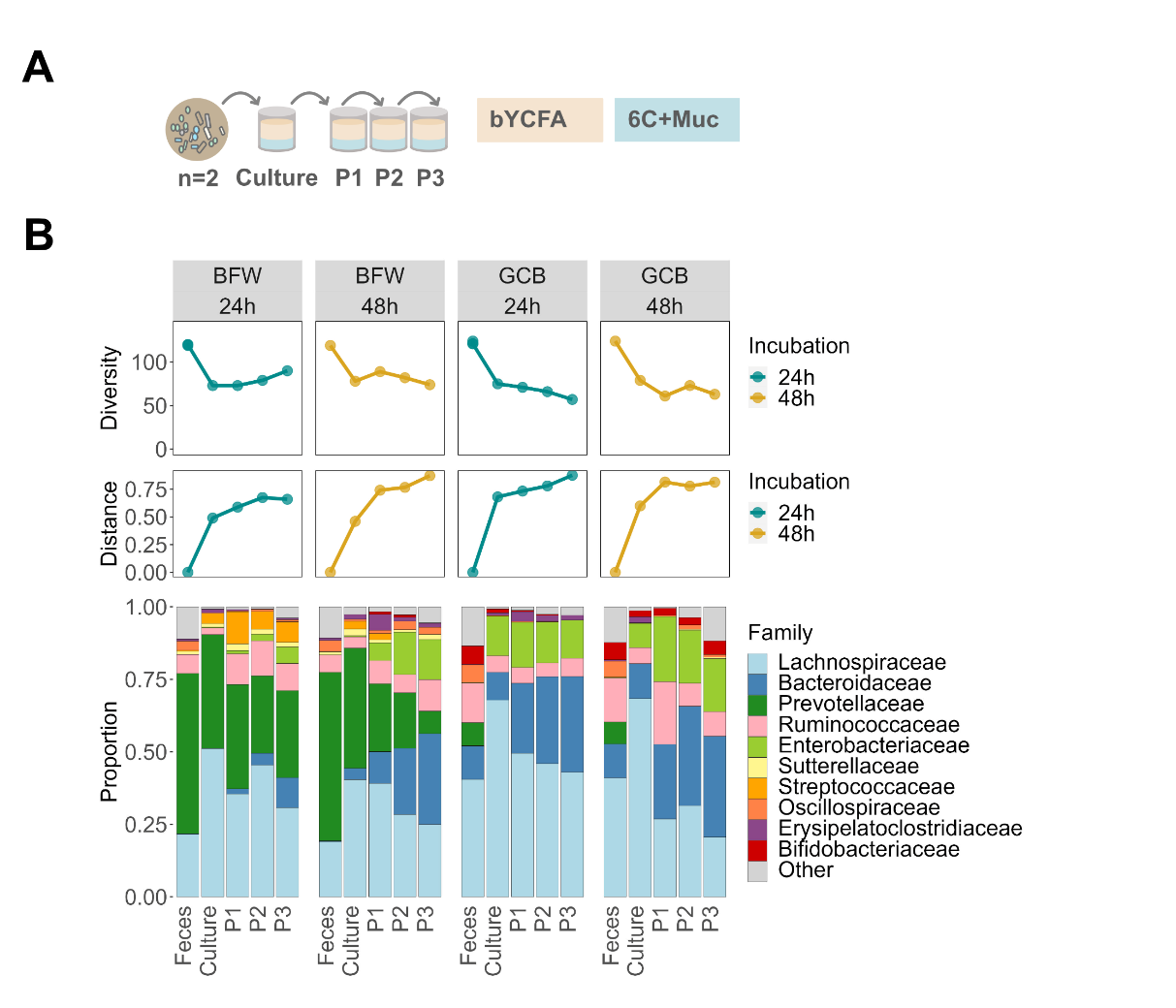


**Figure S7: Characteristics of cultures derived from donor microbiota BFW and GCB repeatedly passaged by re-inoculating 1% (3 passages, 24/48 h, 37°C, donors n=2) into bYCFA with 6C+Muc.**

**A)** Experimental setup for successive passages of *ex vivo* cultures. **B)** Alpha diversity (observed ASVs; top), Bray-Curtis distances between cultures and feces (middle) and community composition represented in taxa bar plots (below). All analyses were performed on pooled samples from three independent replicates for each donor microbiota.


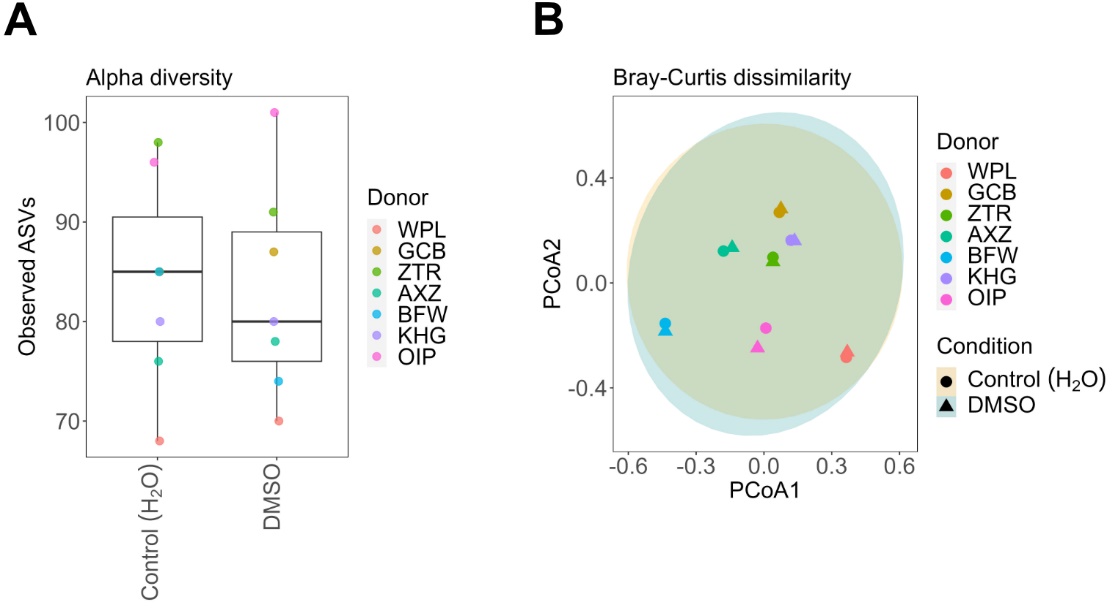


**Figure S8: Characteristics of *ex vivo* cultures grown in bYCFA with 6C+Muc (48 h, 37°C, donors n=7) and exposed to 0.2 % DMSO compared to control (H_2_O).**

**A)** Number of observed ASVs in cultures treated with 0.2% DMSO or H_2_O (control). **B)** Bray-Curtis distances between cultures treated with DMSO (0.2%) and H_2_O visualized in a PCoA plot.

All analyses were performed after 48 h on pooled samples from three technical replicates for each donor microbiota.

**Table S1: The modular components of the final medium and their composition.**

The basal medium, heat-stable and heat-sensitive supplements were prepared based on the respective concentration factor and mixed according to % of the final volume.

| **Component type** | **Composition** | **Concentration factor** | **% of the final volume** |
| --- | --- | --- | --- |
| **Comparison of 96-deepwell plates and Hungate tubes using fecal microbiota** **(Figure 2)** | | | |
| Basal medium | bYCFA | 1.33X | 75% |
| Heat-stable supplement | Dextrin, starch, H_2_O | 4X | 25% |
| **Comparison of 96-deepwell plates and Hungate tubes using pure cultures** **(Figure S4)** | | | |
| Basal medium | bYCFA | 1.33X | 75% |
| Heat-stable supplement | Arabinogalactan, pea fiber, dextrin, xylan, starch, pectin | 4X | 25% |
| **Optimization of C- and N-source composition to maintain *in vivo*-like communities** **(Figures 3, S5)** | | | |
| Basal medium | bYCFA | 1.33X | 75% |
| Heat-stable supplement | Glucose, 6C, 6C+Mucin, 3C, 3C+Mucin, H_2_O, BHI-like, GMM-like | 4X | 25% |
| **Optimization of metabolic profile by additional N-source (Figures 3, S6)** | | | |
| Basal medium | bYCFA | 1.33X | 75% |
| Heat-stable supplement | 6C+Mucin | 8X | 12.5% |
| Heat-stable supplement | N-source, H_2_O | 8X | 12.5% |
| **Treatment of *ex vivo* cultures with dietary fibers** **(Figure 4)** | | | |
| Basal medium | bYCFA | 1.33X | 75% |
| Heat-stable supplement | Dextrin, starch, H_2_O | 4X | 25% |
| **Treatment of *ex vivo* cultures with drugs** **(Figure 4, S8)** | | | |
| Basal medium | bYCFA | 1.33X | 75% |
| Heat-stable supplement | 6C+Mucin | 8X | 12.5% |
| Heat-sensitive supplement | omeprazole, ciprofloxacin, 5-FU, H_2_O | 8X | 12.5% |

**Table S2: The composition of the heat-stable and heat-sensitive supplement solutions.**

The supplement solutions were prepared in a 4- or 8-fold concentrated manner according to Table S1 to reach final concentrations as listed below.

| **Suplement** | **g/L (final concentration)** | **Compound** | **Provider** |
| --- | --- | --- | --- |
| **Heat-stable supplement solutions** | | | |
| Dextrin | 3 | resistant dextrin (NUTRIOSE® FB06) | Roquette, Lestrem, France |
| Starch | 3 | starch (soluble starch from potato) | Sigma-Aldrich |
| Glucose | 3 | glucose | Sigma-Aldrich |
| 6C±Muc | 0.45 | starch (soluble starch from potato) | Sigma-Aldrich |
|  | 0.45 | pectin (pectin from citrus peel; | Sigma-Aldrich |
|  | 0.45 | xylan (xylan from oat spelt) | Angene, London, United Kingdom |
|  | 0.24 | arabinogalactan (from larch wood) | Sigma-Aldrich |
|  | 0.24 | guar | Sigma-Aldrich |
|  | 1.14 | inulin (Orafti GR) | Beneo, Mannheim, Germany |
|  | ± 0.3 | mucin from porcine stomach type II | Sigma-Aldrich |
| 3C±Muc | 1 | starch (soluble starch from potato) | Sigma-Aldrich |
|  | 1 | cellobiose | Sigma-Aldrich |
|  | 1 | glucose | Sigma-Aldrich |
|  | ± 0.3 | mucin from porcine stomach type II | Sigma-Aldrich |
| BHI-like | 3 | glucose | Sigma-Aldrich |
|  | 16.5 | meat extract | Sigma-Aldrich |
|  | 10 | Peptone (of animal tissue) | Neogen, Lansing, Michigan, United States |
| GMM-like | 0.35 | glucose | Sigma-Aldrich |
|  | 0.88 | cellobiose | Sigma-Aldrich |
|  | 0.88 | maltose | Sigma-Aldrich |
|  | 0.88 | fructose | Sigma-Aldrich |
|  | 4 | meat extract | Sigma-Aldrich |
|  | 1 | amicase | Sigma-Aldrich |
| N-source solutions | 8.2 | amicase | Sigma-Aldrich Chemie GmbH, Buchs, Switzerland |
|  | 2.25 | yeast extract | Lesaffre, Marcq-en-Barœul, France |
| **Heat-sensitive supplement solutions** | | | |
| Ciprofloxacin | 0.0166 | Ciprofloxacin - hydrochloride | Sigma-Aldrich |
| Omeprazole | 0.004 | omeprazole (dissolved in 2% DMSO) | Sigma-Aldrich |
| 5-FU | 0.0065 | 5-fluorouracil | Sigma-Aldrich |

**Table S3: Bacterial strains used in this study and corresponding carbon source supplemented for growth.**

Respective fibers were supplemented to reach final 3 g/L, and glucose and organic acids were applied at a concentration of 30 mM. All compounds were purchased from Sigma-Aldrich, except Dextrin (Roquette, Lestrem, France) and xylan (Angene, London, United Kingdom).

| **Genus** | **Species** | **Strain** | **Culture collection** | **Supplemented C-source** |
| --- | --- | --- | --- | --- |
| *Faecalibacterium* | *duncaniae* | DSM 17677 | DSMZ | Glucose |
| *Bacteroides* | *thetaiotaomicron* | DSM 2079 | DSMZ | Glucose |
| *Eubacterium* | *ramulus* | PB-SKBSD | PB | Arabinogalactan |
| *Phocaeicola* | *dorei* | PB-SXBFK | PB | Pea fiber |
| *Phascolarctobacterium* | *faecis* | PB-SDVAP | PB | Succinate |
| *Acutalibacter* | sp. | PB-SMJER | PB | Dextrin |
| *Bifidobacterium* | *adolescentis* | PB-SKRVN | PB | Xylan |
| *Agathobacter* | *rectalis* | PB-SWUPP | PB | Soluble starch |
| *Frisingicoccus* | sp. | PB-SKUSI | PB | DL-Lactate |
| *Lachnospira* | *eligens* | PB-SJATG | PB | Pectin |

DSMZ: German Collection of Microorganisms and Cell Culture GmbH; PB: PharmaBiome AG.
