# Supplementary material for: A flexible high-throughput cultivation protocol to assess the response of individuals’ gut microbiota to diet-, drug-, and host-related factors
Source: ISME Commun. 2024 Mar 12;4(1):ycae035. doi: 10.1093/ismeco/ycae035 (PMC10982853; doi:10.1093/ismeco/ycae035)
Supplement: Supplementary_file_1_Zund_et_al_ycae035 [file supplementary_file_1_zund_et_al_ycae035.docx]

**Supplementary File 1: Protocol for the high-throughput cultivation of gut microbiota and microbes in an anaerobic chamber**

***A) Purpose and Principle***

This protocol was developed to test the responses of individuals’ gut microbiota and pure cultures to various factors of the intestine related to diet (e.g., fibers, proteins, vitamins), host physiology (e.g., pH, oxidative stress, bile salts) or drugs (e.g., antibiotic and non-antibiotic medication).

The protocol consists of the following steps and respective estimated hands-on times (calculated for six donor microbiota and 20 conditions), starting with the preparation of the medium and solutions (**Step 1 ~4 h**) a few days prior to the experiment. This is followed by setting up the anaerobic chamber (**Step 2 ~15 min**) and allowing the medium to equilibrate (**Step 3 ~10 min**) on the day before the experiment. The culture conditions are then finalized (**Step 4 to 6 ~3.5 h**), and the microbes are inoculated (**Step 7 ~30 min**) on the day of the experiment. Finally, sample preparation and analysis are described (**Step 8 ~1 h 20 min**). On the day of the experiment (**Step 4 to Step 7**), it is possible to work alone. However, if the number of fecal donors and/or conditions to be tested is high, it should ideally be done by two people in parallel to ensure that inoculation is performed immediately after the pH adjustment of the basal medium.

Based on the indicated per-step hands-on time, around ten total working hours are required for the full procedure when testing six donor microbiota and 20 conditions. Testing of one instead of six donor microbiota is estimated to be around 6 hours, illustrating that the per-donor testing time decreases as the number of donors increases since the time required for most Steps (i.e., Steps 1-4 and Step 6) does not increase proportionally. Followingly, we highly recommend testing multiple donor microbiota in parallel, with six microbiotas being a technically feasible number. The medium comprises modular parts (the basal medium and heat-stable/sensitive supplements) that are separately customizable, providing high flexibility. Here, the step-by-step protocol was written based on the example of testing fecal cultures’ responses to drugs. In this specific example, the medium is composed of 75% bYCFA (basal medium, 1.33X concentrated), 12.5% 6C+Muc (heat-stable supplement, 8X concentrated), and 12.5% drug solutions (heat-sensitive supplement, 8X concentrated). Depending on the research question, the experimental design can be simplified or expanded to other medium compositions, treatments (e.g., dietary fibers), and physicochemical conditions (e.g., pH).


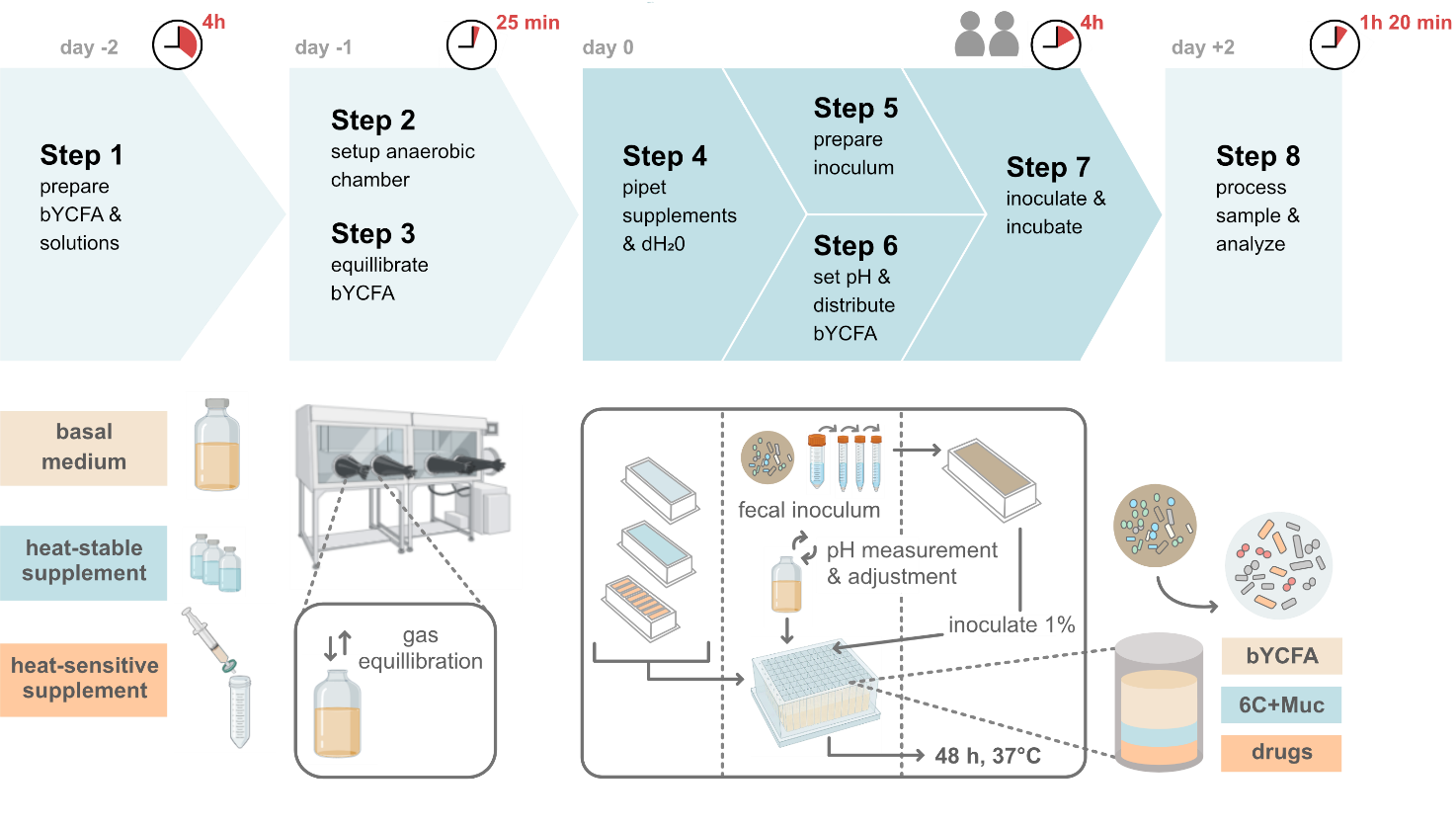


**Figure 1:** Overview of the different Steps of the high-throughput protocol for the cultivation of gut microbiota and microbes with estimated hands-on-times for testing six donor microbiotas and 20 conditions.

***B) Reagent composition and preparation***

*Note: Aerobic solutions can be prepared directly on the bench. The* *anaerobic medium and anaerobic heat-stable solutions are boiled and when cooled down, either CO_2_ or N_2_ can be used (interchangeably, depending on availability) to help remove O_2_ traces. However, it needs to be considered that flushing with CO_2_ (preferred for bicarbonate-buffered media) acidifies the solution, while N_2_ does not (preferred for non-buffered solutions). After transfer to the anaerobic chamber, the medium pH will change again depending on the CO_2_ concentration in the chamber.*

**#1 Vitamin solution (aerobic):** Dissolve 10 mg biotin, 10 mg cyanocobalamin, 30 mg *p*-aminobenzoic acid, 50 mg folic acid, and 150 mg pyridoxamine in 100 mL deionized water (dH_2_O). Cover aliquots with aluminum foil and store them at 4°C (max. 3 months) or -20°C (long-term storage).

**#2 Mineral solution I (aerobic):** Dissolve 3.5 g K_2_HPO_4_ in 1 L dH_2_O. Store at 4°C.

**#3 Mineral solution II (aerobic):** Dissolve 3.5 g KH_2_PO_4_, 6 g NaCl, 6 g (NH_4_)_2_SO_4_, 0.6 g MgSO_4_, and 0.6 g CaCl_2_ in 1 L dH_2_O. Store at 4°C.

**#4 Hemin stock solution (aerobic):** Dissolve 1.25 g hemin from bovine in 5 mL NaOH 5 M and add 20 mL dH_2_O. Cover with aluminum foil and store at 4°C (max. 3 months).

**#5 Volatile fatty acid solution (aerobic):** Within a chemical hood, on ice, mix 17 mL acetic acid (>99.5%), 6 mL propionic acid (>99.5%), 1 mL isovaleric acid (>99%), 1 mL isobutyric acid (>99%), and 1 mL valeric acid (>99%), and complement with 24 mL of 5 M NaOH. Store at room temperature.

**#6 Resazurin stock solution (aerobic):** Dissolve 100 mg resazurin sodium salt in 100 mL dH_2_O. Store at 4°C.

**#7 bYCFA basal media (1.33X concentrated, anaerobic):** The cultivation medium is prepared with a concentration factor (here 1.33X) to account for further addition of (heat-stable/sensitive) supplements (here C-source mix and drug solutions). In an Erlenmeyer flask, mix all nitrogen sources (1 g amicase, 1.25 g yeast extract, 0.5 g meat extract) with 0.1 mL vitamin solution (**Reagent #1**), 150 mL mineral solution I (**Reagent #2**), 150 mL mineral solution II (**Reagent #3**), 0.2 mL hemin stock solution (**Reagent #4**) and 6.2 mL volatile fatty acid solution (**Reagent #5**). Fill with dH_2_O to 750 mL (1.33X concentrated medium) and adjust pH to 7.5 (5 M NaOH). Attach a condenser to the Erlenmeyer flask and boil (10 min) to remove O_2_ on a heating plate with a magnetic stir bar. Cool down to ~60°C and add the remaining components (4 g NaHCO_3_, 1 g L-cysteine HCl), all under constant flushing with CO_2_. After 10 min at ~60°C, fill the medium under constant CO_2_ flushing in DURAN® Pressure plus flasks (size between 250-1000 mL) containing a magnetic stir bar. Close with airtight butyl stoppers and autoclave soon after. Store at room temperature.

*Note: 90 mL of 1.33X basal medium is required per 96-deepwell plate, when using 2 mL of medium per well and 60 wells per plate (outer wells filled with dH_2_O;* **see Step 4***).*

*To maintain fecal cultures similar to the original feces in terms of taxonomic composition, we suggest complementing the basal medium with the C-source mix, 6C+Muc (see heat-stable supplement: 15% starch, 15% pectin, 15% xylan, 8% arabinogalactan, 8% guar gum and 38% inulin and 10% mucin type II)***.**

**#8 Heat-stable supplement, e.g., 6C+Muc (8X concentrated, anaerobic):** Dissolve 369 mg soluble starch, 369 mg pectin from citrus peel, 369 mg xylan, 185 mg arabinogalactan, 185 mg guar gum, 923 mg inulin and 240 mg mucin type II in 100 mL dH_2_O (resulting in an 8X concentrated solution) and adjust to pH 7. Attach a condenser to the Erlenmeyer flask and boil (10 min) to remove O_2_. Cool down under constant N_2_ flushing. After 10 min at ~60°C, fill the solution under constant N_2_ flushing in glass flasks, close with airtight butyl stoppers and autoclave soon after.

*Note: Heat-stable supplements can be flexibly adapted for testing other autoclavable components, e.g., reducing agents, xenobiotics, antibiotics or vitamins. When calculating the final concentrations to use in the plate, consider that a concentrated stock solution should be prepared (in this protocol, supplements are 8X concentrated).*

**#9 Heat-sensitive supplement, e.g., drug solutions (8X concentrated, anaerobic):** Dissolve the desired drug (8X concentrated) in anaerobic dH_2_O (**Reagent #11**) directly in the anaerobic chamber. Sterilize using a 0.2 µm syringe filter.

*Note: Heat-sensitive stock solution can be flexibly adapted. When calculating final concentrations, consider the higher concentration of the stock solution (here, 8X concentrated). When testing difficult-to-solubilize compounds, DMSO*^[[1]](#footnote-2)^ *can be used as a solvent instead (suggested final concentration of 0.2% in the plate).*

**#10 Phosphate buffer for diluting fecal samples (anaerobic):** Mix 190 mL mineral solution I (**Reagent #2**), 290 mL mineral solution II (**Reagent #3**) and 1 mL of resazurin stock solution (**Reagent #6**), adjust to pH 7 and fill up with dH_2_O to 1 L. Attach a condenser to the Erlenmeyer flask and boil (10 min) to remove O_2_. Cool down to ~60°C under constant CO_2_ flushing and add the remaining components (4 g NaHCO_3_, 1 g L-cysteine HCl). Flush with CO_2_ until the solution turns transparent, fill the solution into Hungate tubes (9 mL per tube) under constant CO_2_ flushing, close with airtight butyl stoppers and autoclave soon after.

**#11 Anaerobic dH_2_O (anaerobic):** Heat the desired volume of dH_2_O in a DURAN® Pressure plus flask under constant flushing with N_2_. Boil (10 min), close with an airtight butyl stopper and autoclave soon after.

***C) Step-by-step procedure to cultivate gut microbiota/microbes in the anaerobic chamber using 96-deepwell plates***

**Step 1: Preparation of bYCFA and other solutions**

*Timing: At least two days before the experiment ~4 h*

Prepare reagents as described above, including bYCFA medium (**Reagent #7**; requires **Reagents #1-#5**), heat-stable supplement (e.g., 6C+Muc, **Reagent #8**), heat-sensitive supplement (e.g., drug solution, **Reagent #9**) and phosphate buffer (**Reagent #10;** requires **Reagents #2, #3 and #6**).

*Note: When numerous cultivations over time are anticipated, and repeatability of responses is desired, it is advisable to prepare a large batch of bYCFA media using the same lot of complex components (e.g., yeast extract, meat extract, amicase). Once sterilized, the basal media remain stable and can be utilized in subsequent experiments, thereby minimizing the batch-to-batch variability of results arising from complex components.*

**Step 2: Preparation of the anaerobic chamber**

*Timing: One day before the experiment ~15 min*

The anaerobic chamber should have the following equipment: an incubator, a pH meter, multi-channel pipettes (*e.g*., 100-1200 μL, 10-100 μL), a single-channel pipette (100-1000 μL), a pipette boy and a magnetic plate stirrer. To properly prepare the chamber, i) check gloves for holes, ii) replace the palladium catalysts with freshly rejuvenated Stak-Paks (*i.e.*, incubation at 80 °C overnight), and iii) flush the chamber with gas mix containing H_2_ (5% H_2,_ 10% CO_2_, 85% N_2_). Ideally, the experiment starts with H_2_ above 2.5% (but below 5% due to explosion risks), as H_2_ levels continuously decrease throughout the experiment. All material and reagents (needed for **Steps 3 to 8**), including autoclaved 96-deepwell plates (Ritter Medical), must be placed in the chamber at least 24 h before the experiment to allow proper removal of residual O_2_. When bringing material into the chamber, puncture all packaging material with a needle to allow gas exchange.


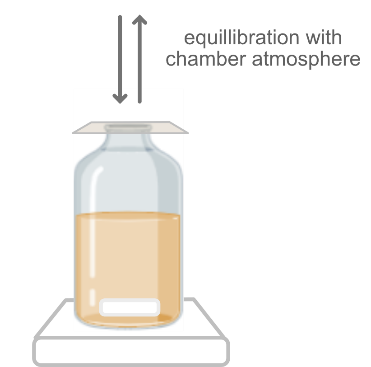
**Step 3: Equilibration of bYCFA in the chamber**

*Timing: One day before the experiment ~10 min*

Place the batch of bYCFA medium (in DURAN® Pressure plus flasks; **Reagent #7**) in the chamber at least 24 h before the experiment. Open the flask, cover it with a Breath-Easier Sealing Film (Diversified Biotech, Dedham, Massachusetts, United States), and place it on a stirrer to let the medium equilibrate with the gas composition of the chamber.

**Step 4: Preparation of the 96-deepwell plates with dH_2_O and compounds to test (e.g., 6C+Muc and drug solutions)**


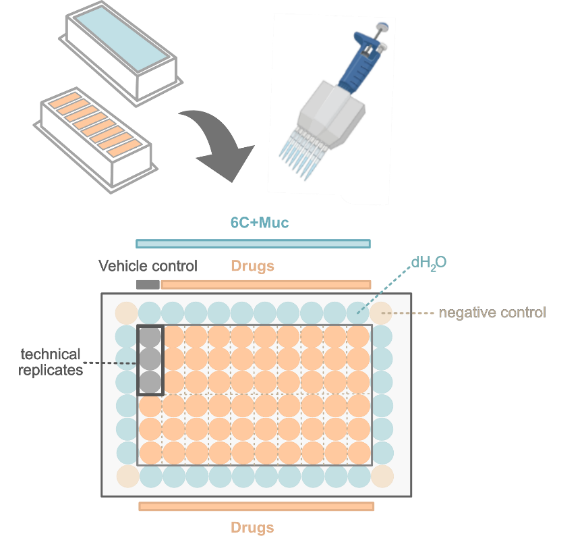
*Timing: Day of the experiment ~80 min (20 min general preparations, 10 min pipetting per plate)*

Important: the subsequent steps present risk of contamination, as the standard Coy anaerobic chamber is not a sterile environment. To mitigate potential contamination: 1) disinfect the surfaces and gloves using a bleach solution (100 mL bleach, 60 mL of 5% acetic acid, 840 mL dH_2_O) or disinfectant tissues (Wetrok Quick & Clean, Kloten, Switzerland), and 2) consistently cover the 96-deepwell plates with the sterile lid between pipetting steps.

Fill the outer wells of the 96-deepwell plate with sterile anaerobic dH_2_O to reduce evaporation of the media during the cultivation and prevent the edge effect^[[2]](#footnote-3)^. Pipet 250 µL of 6C+Muc (heat-stable supplement; **Reagent #8**) and 250 µL of the drug solutions (heat-sensitive supplement; **Reagent #9**) or the vehicle control (e.g., anaerobic H_2_O) into the desired wells using sterile containers and a multi-channel pipette.

*Note: The figure above illustrates an exemplary plate layout to test one donor and 19 drugs plus vehicle control, which, however, can be flexibly adjusted. It is recommended to include contamination control wells (**negative controls) filled with sterile bYCFA supplemented with 6C+Muc. These can be visually monitored for undesired microbial growth. Should the 60 experimental wells be fully occupied, contamination controls can be allocated at the edges of the plate as indicated in the exemplary layout.*

**Step 5: Preparation of the inoculum**

*Note: If the O_2_ level rises while transferring the fecal sample or pure cultures into the chamber, wait until < 20 ppm.*

**5.1. Testing a fecal sample**

*Timing: Day of the experiment ~1.5 h (15 min per sample)*


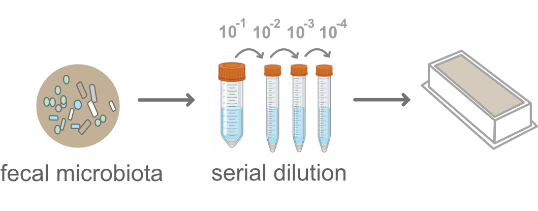
The fecal sample, collected in a plastic recipient containing an AnaeroGen™ 2.5L Sachet (Thermo Scientific™ Oxoid™, Waltham, MA, United States) to reduce the atmospheric oxygen, is brought into the chamber immediately upon arrival in the laboratory. For each donor microbiota, fill one 50 mL and three 15 mL Falcon tubes (for dilutions 10^-1^ to 10^-4^) containing 9 mL of phosphate buffer dilution solution (**Reagent #10**). Take ~1 g fecal sample using a sterile plastic spoon and resuspend it by gently shaking and mixing with the phosphate buffer solution (10^-1^ dilution, 50 mL Falcon tube). Let the main solid particles settle, then stepwise dilute down to the required dilution (10^-4^) by transferring 1 mL into 9 mL phosphate buffer. Transfer the diluted fecal sample into a sterile container (*e.g.,* petri dish or reservoir). Then, the fecal dilution is ready to be inoculated.

**5.2. Testing pure bacterial cultures**

*Timing: Day of the experiment ~15 min*

Typically, a pre-culture is prepared from a glycerol stock by 1-2% (v/v) inoculation of anaerobic bYCFA containing an appropriate C-source in Hungate tubes (37°C, 24 h). On the day of the experiment, OD_600_ of the pre-culture is measured and if needed, the culture is diluted to a defined OD_600_ value (*i.e.,* standardized inoculum). In the anaerobic chamber, transfer the culture into a sterile container (*e.g.,* petri dish or reservoir). Then, the culture is ready to be inoculated.

**Step 6: Setting up starting pH of bYCFA and complementing media in 96-deepwell plates**

*Timing: Day of the experiment ~5 min*


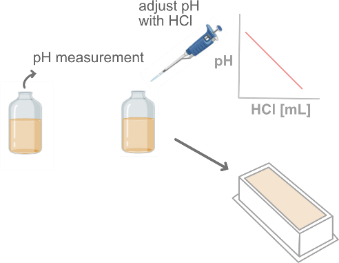
The pH meter should be calibrated on the day of the experiment (use new calibration solutions as the pH is altered when stored in the chamber). First, withdraw a small volume of equilibrated bYCFA medium from the DURAN® Pressure plus flasks (from **Step 3**) to measure initial pH (approximately 7.3 to 7.5). Next, calculate the amount of HCl needed to adjust the pH to the desired value using the formula **(2)**. In brief, the calculations are based on a pre-determined calibration curve generated for bYCFA showing the change in pH upon HCl addition (as described in Figure 1E of the main article), resulting in the linear equation **(1)**.

${pH}_{target}=7.36-39.7*{HCl}_{to add} \left[ mmol/L \right]$ **(1)**

Based on the slope of equation **(1)**, the amount of HCl [mmol/L] to add can be estimated using equation **(2)**.

${HCl}_{to add} \left[ mmol/L \right]=\frac{{{pH}_{target}-pH}_{initial}}{-39.7}$ **(2)**

For example, starting with an initial pH of 7.4 and aiming for a target pH of 6.5, it is necessary to add a total of 0.022 mmol/L of HCl. When using a 3 M HCl stock to adjust the pH of 200 mL bYCFA, a volume of 1.5 µL 3 M HCl should be added.

After adding HCl, re-measure the pH and further adjust if needed. Then, fill 1.5 mL of bYCFA medium into each well of the 96-deepwell plate (pre-filled with supplements). Together with the previously pipetted supplements, the final total volume should be 2 mL.

**Step 7: Inoculation and incubation**

*Timing: Day of the experiment ~30 min (5 min per sample)*

Immediately inoculate the 96-deepwell plates with 20 µL of fresh inoculum (diluted fecal sample (**Step 5.1**) or pure culture (**Step 5.2**)) using a multi-channel pipette. Cover the plate with Breathe-Easier Sealing Film (Diversified Biotech) and incubate (37°C, 48 h).

**Step 8: Sampling and analysis**


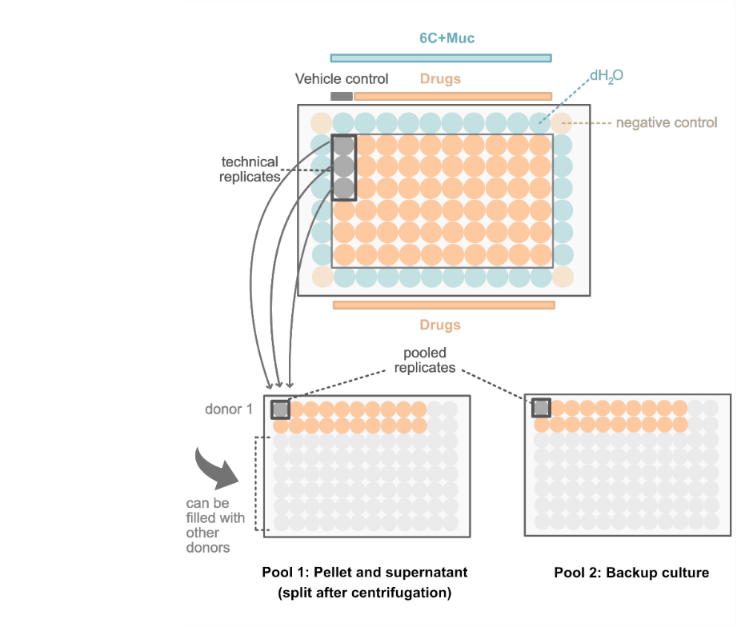
*Timing: 2 Days after the experiment ~1 h 20 min (10 min pooling per plate, 20 min centrifugation, 30 min supernatant and pellet separation)*

2 mL provides enough material for various types of analysis; i) OD_600_ measurements by transferring the culture into a 96-well plate and measuring OD_600_ using an absorbance plate reader (ca. 200 µL), ii) pH measurement by pipetting additional aliquots into well plates (ca. 200 µL) and measure pH (preferably in the anaerobic chamber) using a pH microelectrode (diameter 6 mm; Metrohm AG), iii) metabolite analysis by liquid chromatography using the supernatant (ca. 300 µL) and iv) 16S rRNA amplicon sequencing using cell pellet (ca. 1 to 2 mL).

To collect the supernatant and pellet, centrifuge cultures using a rotor adapted to 96-deepwell plates (5500 rpm, 20 min, 4°C). Then, split the pellet and resulting supernatants by pipetting the latter into another 96-deepwell plate. Both can be stored at -80°C (covered with an adhesive seal) until subsequent sample analysis. For organic acids quantification, *e.g.,* via liquid chromatography (U/HPLC), supernatants are filtered using a 0.2 µm nylon membrane filter, preferably using a plate-based filtration system (AcroPrep™ Filter Plates; VWR International AG). The cell pellet can be resuspended in buffer for subsequent extraction of DNA.

Depending on the experimental design and the number of samples to analyse, pooling the technical replicates can be performed prior to analysis (see Figure). However, it is important to evaluate for each experimental set-up or sample type if pooling is reasonable and define criteria for pooling beforehand. Prepare two pools by transferring 0.5 mL from each replicate into a fresh plate (resulting in 1.5 mL sample volume). One pool can be used for direct analysis and the second sample pool can be stored at -80°C and considered as a backup.

1. *Chaveau, A., et al. (2023). Intestinal permeability and gut microbiota interactions of pharmacologically active compounds in valerian and St. John’s wort. Biomed. Pharmacother.*  [↑](#footnote-ref-2)
2. *Douglas S. Auld, et al. (2020). Microplate Selection and Recommended Practices in High-throughput Screening and Quantitative Biology. Assay Guid. Man.* [↑](#footnote-ref-3)
